# Primary Effusion Lymphoma: A Timely Review on the Association with HIV, HHV8, and EBV

**DOI:** 10.3390/diagnostics12030713

**Published:** 2022-03-15

**Authors:** Chih-Yi Liu, Bo-Jung Chen, Shih-Sung Chuang

**Affiliations:** 1 Division of Pathology, Sijhih Cathay General Hospital, New Taipei City 221, Taiwan; cyl1124@gmail.com; 2 School of Medicine, College of Medicine, Fu Jen Catholic University, New Taipei City 221, Taiwan; 3 Department of Pathology, Shuang Ho Hospital, Taipei Medical University, New Taipei City 221, Taiwan; b8801061@gmail.com; 4 Department of Pathology, School of Medicine, College of Medicine, Taipei Medical University, Taipei 110, Taiwan; 5 Department of Pathology, Chi-Mei Medical Center, Tainan 710, Taiwan

**Keywords:** EBV, effusion-based lymphoma, HHV8, HIV, primary effusion lymphoma

## Abstract

Primary effusion lymphoma (PEL) is defined by the WHO classification as a large B-cell neoplasm without detectable tumor masses. It is universally associated with HHV8, with most cases occurring in the setting of immunodeficiency such as HIV infection, and a poor prognosis. Morphologically, the neoplastic cells range from immunoblastic, plasmablastic, to anaplastic; and phenotypically, most cases express plasma cell but not B-cell markers, i.e., plasmablastic. During the past decade, primary HHV8-negative effusion lymphoma has been reported. Such cases were considered in the WHO classification scheme as effusion-based lymphoma. We performed a systemic review of 167 HHV8-negative effusion lymphomas from the literature and found that only 42% were associated with a fluid overload state, and with low rates of HIV (6%) or EBV (21%) infection. Furthermore, most patients are old (or immunosenescent) with underlying medical conditions/comorbidities, most neoplasms are of B-cell phenotype, and the outcome is more favorable than that of HHV8-positive PEL. These distinctive findings supported our prior proposal of designating these HHV8-negative cases as type II PEL, in contrast to the classic or type I PEL as defined by the WHO. Furthermore, we propose an algorithmic approach for the diagnosis of PEL and its mimickers.

## 1. Background

Lymphomas in effusions may be primary or secondary. Among lymphomatous effusions, secondary involvement by hematologic malignancies is more common than primary lymphomas arising in the body cavities without detectable masses [1,2,3,4]. In secondary cases, lymphomatous effusions more commonly present as a complication of advanced diseases and rarely as an initial presentation [3,5].

Primary effusion lymphoma (PEL) was first described in 1989 as an AIDS-related lymphoma [6]. Nador et al. first proposed PEL as a human herpes virus 8 (HHV8)-associated lymphoma whose main tumor was present in the body cavity fluid in 1996 [7]. In the current updated 4th edition of the World Health Organization (WHO) classification of hematolymphoid neoplasms, PEL is defined as a large B-cell neoplasm usually presenting as serous effusion without detectable tumor masses [8]. It is described to be universally associated with HHV8, also called Kaposi sarcoma-associated herpes virus (KSHV), and most often occurs in the setting of immunodeficiency such as those with HIV infection. Up to 80% of the PEL patients had a history of HIV infection [9]. Coinfection with Epstein-Barr virus (EBV) was observed in approximately 80% of PEL cases, which could be absent in elderly patients in HHV8-prevalent regions [8,10]. In the current WHO scheme, readers are advised to distinguish PEL from the rare HHV8-negative effusion-based lymphoma (EBL), which is morphologically similar to PEL and has been described in patients with fluid overload states [8].

In contrast to the current WHO definition of PEL occurring most often in patients with HIV infection, in our prior study from Taiwan we found that patients with PEL were more frequently HIV-unrelated [10]. Furthermore, a subset of HHV8-unrelated PEL, or EBL according to the WHO, with distinct clinicopathological characteristics, including those with or without fluid overload states, has been identified in recent years [4,10,11,12,13,14,15,16,17]. These HIV- and HHV8-unrelated effusion lymphomas occurring primarily in the body cavities might be considered a separate entity or be within a wider spectrum of PEL as currently defined in the WHO classification.

In this review, we discuss the new insights of PEL, focusing on the diagnostic challenges in an attempt to clarify the boundaries between PEL, EBL, and related lymphoproliferations.

## 2. The Association of Immunodeficiency with PEL

In their original report of PEL, Nador et al. described the association of HHV8/KSHV in an unusual subset of AIDS-related lymphomas that grow mainly in body cavities [7]. Apart from AIDS, PEL has also been reported in association with other immunodeficiency conditions, namely, iatrogenic immunodeficiency after solid organ transplantation, cirrhosis, and cancers [18].

Elderly people infected with HHV8 are at an increased risk of PEL due to aging, or immunosenescence. According to Rossi et al., this involves a dual alteration of the immune system that involves a chronic increase in the pro-inflammatory status (so-called inflammaging) and decreased cell-mediated immunity (immunosenescence), both may promote HHV8 replication and pathogenicity [19]. Similar to AIDS patients, most of the HIV-negative patients in the study by Rossi et al. had lymphocytopenia or a low CD4 count in the peripheral blood, which might be encountered in elderly patients and could be considered a minor immunosuppression. These data provide an interesting perspective to age-related changes in immune function that may be a risk factor for active HHV8 infection, despite the absence of HIV [19]. However, in other studies of HIV-negative PEL, CD4 count was not reported [10,20,21].

## 3. The Clinical Spectrum of Immunodeficiency States Associated with PEL

### 3.1. HIV

PEL is considered one of the rare types of AIDS-related non-Hodgkin lymphoma, including diffuse large B-cell lymphoma (DLBCL), Burkitt lymphoma (BL), primary central nervous system lymphoma, and PEL. DLBCL and BL are the main tumor types, with better outcomes than PEL [22,23]. PEL represents 1–4% of HIV-related lymphomas, usually with a decreased number of CD4^+^ T-cells at diagnosis [24]. The median age at diagnosis is 42 to 45 in HIV-infected patients, much younger than the 7th to 8th decades in the general population without HIV infection [10,22,24]. HIV infection is an important factor in the pathogenesis of PEL. In the HIV-positive patients, HHV8 infects human B cells (and other cells) and encodes viral oncogenes such as viral interleukin (vIL)-6, the viral homolog of the Fas-associated death domain-like IL-1β converting enzyme inhibitory protein [9]. Viral dysregulation of human B-cells is likely to play a role in the development of PEL. Chronic antigen stimulation and overproduction of cytokines, including cellular IL-6 and IL-10 in HIV-positive patients, also play potential pathogenetic roles in PEL, similar to that occurs in other B-cell non-Hodgkin lymphomas [9].

The HIV and other viral infections have been associated with a variety of hematologic complications resulted from “cytokine storm”, such as multicentric Castleman’s disease (MCD) [25]. HHV8-associated MCD is caused by uncontrolled infection with HHV8 leading to a cytokine storm driven primarily by excessive production of human interleukin-6 and viral interleukin-6 [26,27,28]. Patients with MCD may also develop HHV8 germinotropic lymphoproliferative disorder, a monotypic proliferation of HHV8+ plasmablasts that are usually coinfected with EBV [25]. Cytokine storm underlies several hematologic syndromes and has recently emerged as a key factor in patients with severe infection by the novel Coronavirus disease 2019 (COVID-19) [26,27,28].

Recent data also suggests that HIV promote lymphomagenesis, not only indirectly as a consequence of its ability to sustain a chronic B-cell activation but also directly through HIV-encoded proteins, particularly HIV p17 protein variants with an enhanced B-cell clonogenic activity [29].

### 3.2. Iatrogenic Immunodeficiency

Patients with iatrogenic immunodeficiency show a high prevalence of lymphoproliferative disorders (LPD). The prototype is post-transplant lymphoproliferative disorders (PTLD) occurring as a consequence of immunosuppression in a recipient of a solid organ or bone marrow transplantation. The other iatrogenic immunodeficiency-associated LPD arises from patients with autoimmune diseases, such as rheumatoid arthritis (RA) treated with immunosuppressive agents, most commonly methotrexate [30].

PTLDs constitute a wide spectrum of EBV-driven or EBV-negative LPDs. In the current 2017 WHO classification, PEL is not listed as an entity in the spectrum of PTLDs [31]. In 1995, Jones et al. reported the first case of PEL occurring 94 months after heart transplantation [32]. A recent systemic review identified 13 cases of post-transplant PEL, including kidney (*n* = 6), heart (*n* = 3), liver (*n* = 2), and one each of intestine and bone marrow [33]. Furthermore, Kaposi sarcoma (KS) occurred in association with PEL in four of the cases with solid organ transplantation; yet most of these patients were HIV-negative [33]. The prognosis of post-transplant PEL is poor, with most cases die within a year [33].

Iatrogenic immunosuppression may cause reactivation of HHV8, leading to an uncontrolled expansion of latently infected endothelial cells or mature post-germinal center B-cells. HHV8-related diseases that occur in the post-transplant setting may be the result of reactivation of a pre-existing HHV8 infection in the recipient, or of HHV8 transmission from HHV8-seropositive donors [34]. Recent data suggested that the risk of post-transplant KS is mainly due to HHV8 reactivation rather than organ-related HHV8 transmission from the donors [33,35]. The same phenomenon may be hypothesized for post-transplant PEL [33,34]. Unlike EBV-associated PTLDs, long-term immunosuppression is important in post-transplant PEL that frequently develop several years after transplantation. Furthermore, post-transplant PEL is usually negative for EBV coinfection, unlike in HIV-positive typical PEL in which the tumor cells are frequently EBV-positive [33].

To date, there were only two cases of PEL developing in patients with iatrogenic immunodeficiency other than post-transplantation. The first case was reported by Perier et al., a patient with retinal vasculitis treated with Rituximab due to a suboptimal response to cyclophosphamide. That patient was negative for HIV and the lymphoma was diagnosed as solid PEL four months after the first dose of Rituximab [36]. However, that patient had enlarged lymph node(s) and the accompanying figure showed a solid tumor, suggesting that this was either not a genuine PEL (i.e., lymphoma cells in the effusion but devoid of any mass lesion) or an extra-cavitary PEL. The second reported case developed PEL during the course of anti-synthetase syndrome (an immune-related multi-organ chronic disorder) treated with tacrolimus (an immunosuppressant) [37]. Interestingly, to date there is no report of PEL occurring in patients with RA or autoimmune diseases treated with methotrexate. Therefore, we conclude that PEL in iatrogenic immunodeficiency is rare, with the majority occurring in post-transplant setting and those other than organ transplantation are extremely rare.

### 3.3. Immunosenescence

PEL has been reported in HIV and HHV8-negative elderly patients without immunodeficiency [10,19,20,38]. In our previous study of 26 Taiwanese patients with a median age of 76.5, only one case was HIV-positive, while the others were either negative or presumed negative based on the clinical setting [10]. There was also no history of congenital immunodeficiency, marrow or organ transplantation, or immunosuppressant/immunomodulatory medication [10]. Therefore, immunosenescence might play a major role in the development of PEL in these patients, as in the cases of EBV-positive DLBCL, which occurs more frequently in the elderly [10].

Immunosenescence refers to the decreased/downregulated ability of an aging immune system to produce an appropriate and effective response to immunological challenges. It is a complex biological process involving both the innate and adaptive immune systems. Immunosenescence leads to a change from loss of diversity in the T-cell receptor repertoire to an increase in the number of exhausted CD28-negative T-cells, and profound functional changes in the subpopulations of CD4-positive T-cells. These alterations favor the gradual development of a state of chronic inflammatory process called “inflammaging” [39]. As immunosenescence promotes chronic infection and leads to defects in anti-cancer immunity, it could be one of the key factors to explain the link between aging and lymphomagenesis [40]. Immunosenescence may result in a propensity to reactivation or infection by lymphotropic viruses such as EBV, HHV8, and HTLV-1, with EBV as the major player in neoplastic lymphoproliferations in elderly patients [39].

## 4. The Association of Viral Infection with PEL

### 4.1. HHV8/KSHV Infection

The spectrum of HHV8-positive lymphoproliferative disorders is broad, ranging from reactive lymphoproliferation, multicentric Castleman disease, classic PEL, HHV8-positive large B-cell lymphoma, to HHV8-positive germinotropic lymphoproliferative disorder, often with overlapping features among different HHV8-positive diseases [41,42,43]. Classical PEL as currently defined in the WHO classification scheme occurs usually in HIV-positive patients with lymphomatous effusion cells positive for HHV8, indicating that HHV8 infection plays a key role in lymphomagenesis [44]. Similar to other herpes viruses, HHV8 infects host cells in two different forms: latent and lytic infection. Five latent gene products, namely, latent nuclear antigen (LANA)-1, LANA-2/viral interferon regulatory factor (IRF)-3, viral homolog of cyclin D (v-Cyclin), viral homolog of FLICE-inhibitory protein (v-FLIP), and Kaposin (K12), are believed to play a significant role in the development of PEL and are involved in lymphomagenesis [24]. Among these, LANA-1, also called ORF73, is the most important latent protein and its detection by immunohistochemistry is very useful to recognize infected cells and to establish the diagnosis of HHV8-related lymphoproliferative lesions [45]. It is worthy to note that HHV8 can infect lymphoid cells and other cell types such as endothelial cells and persists lifelong in a latent form. With decreased immune status such as HIV infection or immunosenescence, HHV8 may reactivate the replicative lytic cycle that produces viremia [39,45].

In addition to the classic HHV8-related PEL, there are reports of sporadic cases and small series of HHV8-unrelated effusion lymphomas. Supplementary Table S1 lists all the HHV8-negative PEL cases in the English literature from 1996 to 2021 that we reviewed [4,7,10,11,12,13,14,16,17,38,46,47,48,49,50,51,52,53,54,55,56,57,58,59,60,61,62,63,64,65,66,67,68,69,70,71,72,73,74,75,76,77,78,79,80,81,82,83,84,85,86,87]. There are a total of 167 cases, with the majority (92.2%, *n* = 154) being large cells with a B-cell phenotype, with rare cases of plasmablastic (3.0%; *n* = 5), T-cell (1.0%; *n* = 2), and indeterminate phenotype (3.6%; *n* = 6). In Taiwan, there was a low prevalence of association with HHV8, accounting for only 32% of PEL cases in our prior study [10]. HHV8-negative or HHV8-unrelated PEL often occur in elderly immunocompetent patients, and only 42% of these cases were associated with chronic fluid overload [10,11,12,13,16]. In contrast to PEL patients in the West, HIV and HHV8 were negative in the vast majority of PEL patients in East Asian countries such as Taiwan, Japan, and Korea [9,10,16,46,88]. Alexanian et al. suggested that HHV8-negative PEL is a distinct entity with characteristic clinical and pathological features different from HHV8-positive PEL [13].

In 2018, based on our prior study and that of others, we proposed to name HHV8-positive cases as “classic” or “type I” PEL and HHV8-negative cases as “type II” PEL, in an attempt to stress the difference of the latter from the WHO-defined PEL, which is universally positive for HHV8 (Table 1) [10]. Table 2 lists the difference of these two types of PEL.

### 4.2. The Association of EBV with PEL

Classic PEL arising from HHV8-infected B-cells are frequently (65–80%) co-infected with EBV (Table 1). Hu et al. reported concurrent EBV infection in 65% (39/60) PEL cases, including 75% (36/48) HIV-positive and 38% (3/8) HIV-negative patients, respectively, indicating a possible synergistic interaction between EBV and HHV8 in the pathogenesis [9]. EBV encodes six nuclear transformation-associated proteins (EBV nuclear antigen (EBNA) 1–6) and immortalizes the infected B-cells. Similar to Burkitt lymphoma (BL), the tumor cells of PEL express EBNA-1 but not LMP-1 and EBNA-2, indicating type I latency [24].

Although EBV coinfection is identified in most cases of classic HHV8-positive PEL, the precise pathogenic role of EBV coinfection in the development of PEL is not fully understood. Kobayashi at el. reported that EBV co-infection is identified in 65.6% (80/122) of classic HHV8-positive PEL; in contrast to 33% (11/33) in HHV8-negative type II PEL (or EBL) [13,62]. Wu et al. reviewed 55 cases of HHV8-unrelated PEL and found that EBV was positive in 30% (16/53) cases [12]. Xiao et al. reported EBV infection in 28% of HHV8-unrelated, type II PEL cases, while many patients also had an underlying medical condition leading to fluid overload [11]. Interestingly, EBV-negative PEL cases most often clustered in HIV-negative elderly patients from HHV8 endemic areas [45]. All these findings argue against a key role of EBV in the development of PEL, especially among the HHV8-unrelated type II PEL.

## 5. The Association of Underlying Medical Conditions in Patients with PEL

As mentioned previously, HHV8-negative type II PELs often occur in elderly, immunocompetent patients [11,12,13,16]. Although HHV8-negative PELs are cytomorphologically similar to classic HHV8-related PEL, such cases are clinically distinct from those with classic PEL (Table 2). These patients are generally HIV-negative and up to half of the cases might have underlying medical conditions, such as by liver cirrhosis and heart failure, leading to fluid overload state [10,12,13,38,45,89]. Wu et al. reviewed 55 cases of HHV8-negative PEL and found liver cirrhosis and heart disease in 10 (18%) and 9 (16%) cases, respectively [12]. Alexanian et al. reported that more than half of the patients had a documented medical condition/comorbidities, either predisposing to or immediately causing a fluid overload state, supporting the concept that the lymphoma may in fact be secondary to a pre-existing effusion [13].

Among the 167 cases of HHV8-negative PEL that we reviewed from the English literature, medical history was available in 129 (77%) cases, with 42.6% (55/129) patients having specific medical conditions including liver cirrhosis (13.1%), renal dysfunction/end-stage renal disease (11.6%), heart failure (10.0%), prior cancer/leukemia (6.2%), and post-transplantation (1.6%), as listed in Table 3. Fluid overload status was present in 41.7% (58/139) patients. Unlike classic PEL, these patients were mostly immunocompetent and their PELs were HHV8-negative, in which fluid overload status or other underlying medical conditions might be related to the lymphomagenesis. In brief, effusion, chronic inflammation, or altered immune status in immunosenescence may create ideal body conditions for lymphomagenesis. These distinctive features led us to propose type II PEL for these HHV-negative cases, in contrast to the classic HHV8-positive, type I PEL [10].

Earlier studies have indicated a possible association between hepatitis C virus (HCV) infection and HHV8-unrelated PEL [11,12,13]. Kobayashi et al. reported that 7 (33%) of their 21 HIV-negative patients with HHV8-unrelated PEL tested positive for HCV [62]. Wu et al. reviewed 40 HHV8-negative PEL cases and found that 25% (*n* = 10) cases were positive for HCV [12]. Interestingly, most HCV-associated, HHV8-unrelated PEL cases involved the peritoneum, which might be related to liver cirrhosis caused by HCV infection [12]. However, in a recent large study from Japan, there is only one HCV-positive case among 61 patients with HHV8-negative PEL [16]. In brief, the association between HCV and HHV8-unrelated type II PEL remains elusive. The role of HCV does not appear as crucial as that of HHV8 in the pathogenesis of PEL.

## 6. What Is Effusion-Based Lymphoma (EBL)?

In the current WHO hematolymphoid book, we are advised that classic PEL should be distinguished from the rare HHV8-negative EBL that is morphologically similar to PEL and has been described in patients with fluid overload states [8]. In the literature, miscellaneous terms such as PEL-like EBL, HHV8-unrelated PEL, and primary HHV8-negative EBL have been coined to describe these cases. The terms “PEL” and “EBL” are confusing in usage in the literature, and sometimes these names seem to have been used interchangeably. As the term “primary effusion lymphoma” is self-explanatory to indicate a primary lymphoma presenting as effusion and is easy to understand. In contrast, the term “effusion-based lymphoma” is a bit obscure and is not easily distinguishable from PEL. Accordingly, in 2018 we proposed to call the HHV8-positive cases “classic” or “type I PEL”, and designate the HHV8-negative cases, including so-called EBL in the WHO scheme, as “type II” PEL [10]. As shown in Table 2, these type II PEL patients are usually elderly and immunocompetent, not infected with HIV, and their tumor cells are usually of B-cell phenotype, and with a lower rate of EBV infection, in contrast to classic HHV8-positive PEL.

## 7. Classification of PEL: Morphology and Immunophenotype

**Figure 1:**
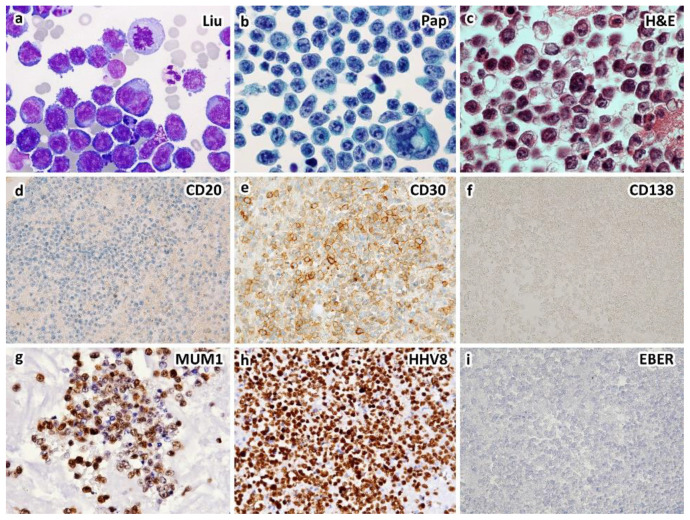
A case of HIV-unrelated, HHV8-positive primary effusion lymphoma occurring in a 72-year-old immunocompetent man who presented with bilateral pleural effusions. Cytological smears show large, atypical lymphocytes with centroblastic, immunoblastic, plasmablastic, and anaplastic morphology by Liu (**a**) and Papanicolaou (**b**) stains. Cell block section shows large, atypical lymphocytes with nucleoli and occasional binucleation ((**c**), H&E stain). Immunohistochemically, the atypical lymphocytes express CD30 (**e**), CD45, HHV8-LANA1 (**h**) and IRF4/MUM1 (**g**), but not CD3, CD19, CD20 (**d**), CD79a, CD138 (**f**), PAX5 or ALK. Ki-67 proliferation index is up to 70%. EBER in situ hybridization is negative (**i**). Primary effusion lymphoma is diagnosed. Serum HIV test is negative. The patient does not have underlying immunodeficiency ((**a**–**c**), ×1000; (**d**–**i**), ×400 magnification).

PEL is typically diagnosed on the basis of effusion cytology. Morphologically, neoplastic cells from classic PEL range from large immunoblastic or plasmablastic cells to cells with more anaplastic morphology (Figure 1) [9,18,45,89,90]. Binucleated or multinucleated cells resembling Reed-Sternberg cells might be present. The nuclei of these cells are round or irregular in shape, with prominent nucleoli. The cytoplasm can be deeply basophilic with vacuoles. Poorly defined perinuclear hofs are often observed [24]. Plasmablastic cells are used to describe key cells in classic PEL [45]. Morphologically, plasmablastic cells are large in size with eccentrically located nuclei, prominent nucleoli, and basophilic/amphophilic cytoplasm [91].

**Figure 2:**
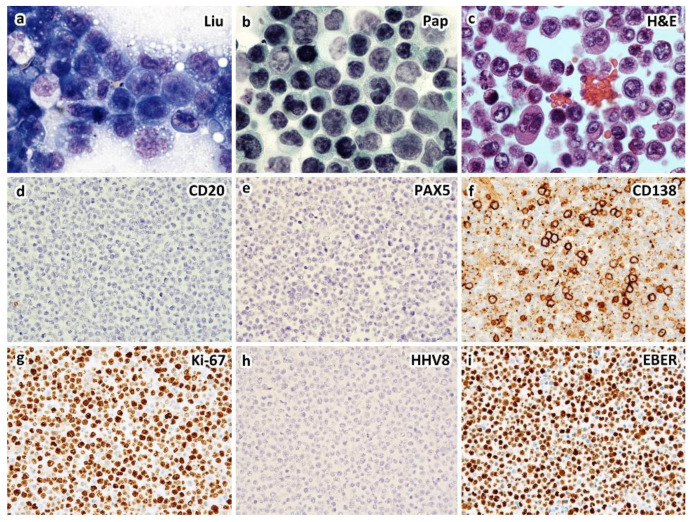
An example of HIV-unrelated, HHV8-negative (type II) PEL of plasmablastic type in a 61-year-old man who presented with left pleural effusion and ascites without solid tumor by CT scans. Cytological smears of the left pleural effusion and ascites show similar features of large, atypical lymphocytes with anaplastic morphology and intracytoplasmic vacuoles by Liu stain (**a**) and Papanicolaou stain (**b**). Cell block section shows large, atypical lymphocytes with occasional Hodgkin/Reed–Sternberg-like cells ((**c**), H&E stain). Immunohistochemically, the atypical lymphocytes express CD138 (**f**), EMA and IRF4/MUM1, but not CD3, CD19, CD20 (**d**), CD30, CD45, CD56, CD79a, PAX5 (**e**), HHV8-LANA1 (**h**), kappa, or lambda light chains. Ki-67 proliferation index is greater than 90% (**g**). EBER in situ hybridization is positive (**i**). Interphase fluorescence in situ hybridization reveals rearrangements of BCL2 and MYC, but not BCL6. Serum HIV assay is negative ((**a**–**c**), ×1000; (**d**–**i**), ×400 magnification).

**Figure 3:**
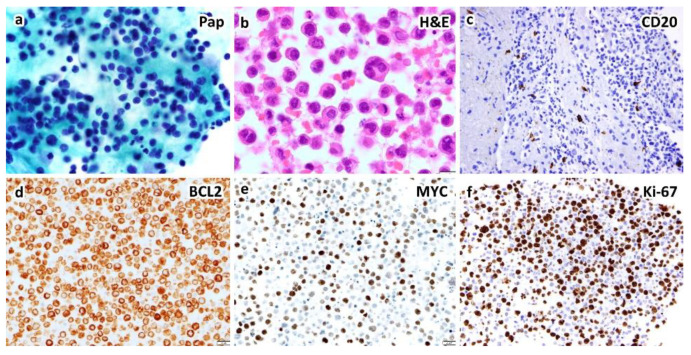
An example of HHV8-negative type II PEL (or effusion-based lymphoma) of indeterminate (B-cell vs. plasmablastic) phenotype in a 59-year-old woman presenting with pleural effusion without hepatosplenomegaly or lymphadenopathy by CT scans. Cytological smears show large, atypical lymphocytes by Papanicolaou stain (**a**). Cell block section shows anaplastic cells and occasional Hodgkin/Reed–Sternberg-like cells ((**b**), H&E stain). Immunohistochemically, the atypical lymphocytes express BCL2 (**d**), BCL6, IRF4/MUM1 and MYC (**e**), but not CD3, CD10, CD20 (**c**), CD30, CD138, cyclin D1, or HHV8-LANA1. Ki-67 proliferation index is up to 70% (**f**). EBER in situ hybridization is negative ((**a**,**b**), ×1000; (**c**–**f**), ×400 magnification).

Most HHV8-unrelated type II PEL demonstrate cytological characteristics ranging from immunoblasts to highly pleomorphic cells, which resemble those commonly found in classic PEL, yet these effusion lymphomas are usually of B-cell phenotype (Figure 2) [10,11,12,38]. In rare cases, the phenotype in terms of B-cell vs. plasmablastic is indeterminate (Figure 3). Unlike classic or type I PEL, HHV8-unrelated type II PEL occasionally reveal small to medium-sized tumor cells, either centroblast-like or Burkitt-like cytomorphology [7,12,18,38]. The broad cytopathological spectrum of type II PEL poses difficulties in diagnosis.

Classic PEL shows a plasmablastic phenotype with neoplastic cells express activation and plasma cell-related markers such as CD30, CD38, VS38c, CD138, and IRF4/MUM1 and usually lack pan-B markers such as CD19, CD20, CD79a, and Pax-5 (Figure 1 and Table 2). Expression of CD45/LCA and epithelial membrane antigen (EMA) is commonly seen [9,24,44,45]. The tumor cells are positive for HHV8-associated protein LANA-1 (typically nuclear and granular), which is useful for the diagnosis of PEL [24,44,45,92]. Surface and cytoplasmic Ig is absent, in contrast to the cytoplasmic IgM lambda expression by the tumor cells of HHV8-positive DLBCL, NOS [45,91]. In situ hybridization for EBV-encoded mRNA (EBER) was positive in about 65–80% PEL cases [8,62]. EBV-LMP1 is variable with absent or low expression in PEL, indicating a restricted latency pattern of EBV infection [91,92].

Rare cases of HHV8-positive lymphoma with features similar to PEL can present as tumor masses and are considered to represent an extracavitary or solid variant of PEL. Compared with classic PEL, the extracavitary or solid variant of PEL had a significantly lower expression rate of CD45, but more frequent expression of CD20, CD79a, and CD138 [93].

It is important to distinguish HHV8-negative PEL from classic PEL because these two appear to differ in pathogenesis, morphological and immunophenotypic characteristics, clinical behavior, and prognosis [12]. Table 2 lists the pertinent clinicopathological features between classic (type I) and HHV8-negative (type II) PEL. The majority of tumor cells in HHV8-negative PEL express pan B-cell markers and do not express activation marker or plasma cell-associated antigen (Figure 2 and Table 2). Our literature review revealed that EBV infection was less common in HHV8-negative PEL (21.0%; 30/143) than in classic HHV8-poisitive PEL (65–80%).

Based on a literature review of 256 cases of PEL, we found in our previous study that *MYC* rearrangement was less frequent in classic than in type II PEL (3% vs. 29%) [10]. A subset of PEL lacks *MYC* translocations, but has deregulated MYC protein expression due to the activity of some HHV8-encoded latent proteins (LANA-1 and vIRF3/LANA-2) [45]. Furthermore, *BCL2* and *BCL6* are rarely rearranged in classic PEL, which could be explained by the different pathogenesis in terms of with or without HHV8 infection [10,91].

In 1998, Ichinohasama et al. proposed a three-tier classification system for effusion lymphomas based on HHV8 and *MYC* status: type I PEL (HHV8 positive, germline *MYC*), type II PEL (HHV8 negative, rearranged *MYC*), and type III PEL (HHV8 negative, germline *MYC*) [83]. Most of their types I and III cases were patients with HIV infection [83]. The subsequent 2001 WHO classification defined PEL as a large B-cell neoplasm universally associated with HHV8, equivalent to type I PEL as defined by Ichinohasama et al. [94]. In 2008, Carbone and Gloghini further classified effusion lymphomas using a constellation of features that included cytomorphology, EBV, HHV8, and *MYC* status into PEL, extranodal large cell lymphoma (HHV8-unrelated PEL-like lymphoma), and extranodal BL [18]. In their classification scheme, the presence or absence of *MYC* translocation was used to classify two different types of HHV8-negative PEL. They suggested that HHV8-unrelated PEL with *MYC* translocations should be classified as a special variant of BL [18,83]. However, alterations in the *MYC* gene and expression of the MYC protein have been identified in aggressive B-cell lymphomas other than BL, such as DLBCL, plasmablastic lymphoma, and ALK-positive large B-cell lymphoma. Secondary involvement of the serous cavities by BL is common, whereas there are only extremely rare examples of BL that presented initially as PEL [4]. We suggest that HHV8-unrelated PEL with *MYC* gene rearrangement should not be considered as extranodal BL.

## 8. Diagnostic Approach for PEL

Serous effusion is a frequent complication of lymphoma progression. Lymphomatous effusion is usually not a diagnostic challenge in patients with a known history of lymphoma; however, it can be particularly challenging when a lymphomatous effusion is the initial presentation. Since ancillary studies, such as immunocytochemistry, flow cytometric immunophenotyping, cytogenetics, and molecular tests, are crucial for diagnosis and classification of lymphomas, sufficient cytology samples are mandatory [11,95]. Please refer to our prior diagnostic algorithm incorporating clinical history, cellular size, cytological atypia, and ancillary studies for the diagnosis of lymphocyte-rich effusions [95].

**Figure 4:**
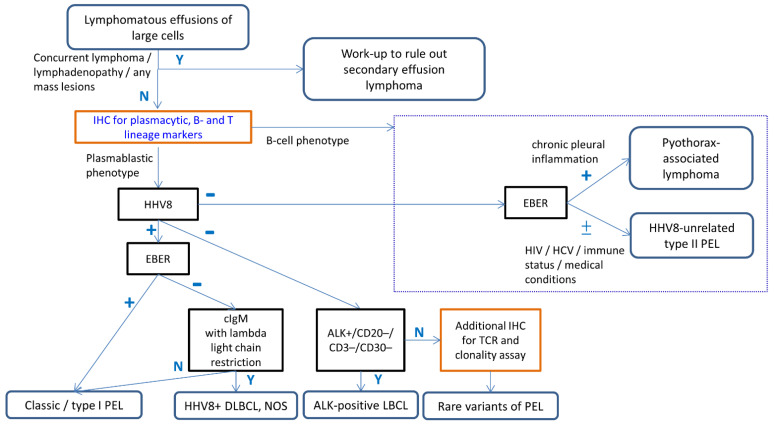
Algorithmic approach incorporating ancillary studies for the diagnosis of PEL. The initial step is the assessment of clinical history of concurrent lymphoma or lymphadenopathy to exclude secondary effusion lymphoma. The proposed initial panel for suspicious PEL includes one marker for each of B, T, and plasma cell lineages. After determining the cellular lineages (plasmablastic vs. B-cell phenotype), HHV8 immunostain and EBER should be performed in all cases of large cell-predominant lymphomatous effusion. When encountering indeterminate examples, a large panel of B, T, and plasma cell markers, as well as clonality assay will be helpful in determining cellular lineages. Careful clinicopathological correlation, including HIV infection, history of immunodeficiency, chronic pleural effusion, other medical conditions, and additional ancillary studies, is needed for the differential diagnosis of PEL with its mimickers. Abbreviations: Y; Yes, N; No.

The morphological spectrum of PEL is wide, including plasmablastic, immunoblastic, and anaplastic features. Classic or type I PEL is characterized by plasmablastic phenotype. However, as there is significant overlap between plasmablastic vs. immunoblastic morphology, immunophenotyping is mandatory for classification. Figure 4 depicts our diagnostic approach for lymphomatous effusion comprising of large neoplastic cells. The proposed initial panel includes one marker each for B, T, and plasma cell lineages, i.e., CD3, CD20, and CD138, respectively. Most of the non-T lineage cases could be triaged into either B-cell or plasmablastic phenotype. If the tumor cells are negative for both CD20 and CD138, additional B-cell markers and plasma cell-related markers should be applied. The additional B-lineage markers include PAX5, OCT2, and BOB1. The other plasma cell related markers include CD38, interferon regulatory factor-4 (IRF4)/MUM1, PR domain zinc finger protein-1 (PRDM1), and/or X-box binding protein-1 (XBP1). CD19 is expressed in neoplastic B-cells and normal/reactive plasma cells, but negative in neoplastic plasmacytic/plasmablastic tumor cells, including classic PEL [96,97].

In a very recent publication on classic PEL from 19 HIV-infected patients, Calvani et al. reported that the neoplastic cells in 53% (*n* = 10) cases expressed CD3, with rare examples positive for CD2, CD4, or CD5 [96]. Such aberrant expression of T-cell markers is a diagnostic pitfall in the diagnosis of PEL. When encountering such atypical cases, a large panel of B, T, and plasma cell markers will be needed. Clonality assay for rearrangements of B or T-cell receptor genes might be helpful in determining cellular lineages.

After determining the cellular lineages (plasmablastic vs. B-cell phenotype), HHV8 immunostain and EBER should be performed in all cases of large cell-predominant, non-T lineage, lymphomatous effusion. Careful clinicopathological correlation, including HIV infection, history of immunodeficiency, chronic pleural effusion, other medical conditions, and additional ancillary studies are needed for challenging cases.

In plasmablastic tumors, the neoplastic cells express plasma cell-related antigens, with frequent loss of B-cell markers [91]. Table 4 lists the major differential diagnoses of the tumors with plasmablastic phenotype, including plasmablastic lymphoma, plasmablastic plasmacytoma/multiple myeloma, PEL and its extra-cavitary variant, ALK-positive large B-cell lymphoma, and HHV8-positive DLBCL, NOS. The most important differential diagnostic clue among these plasmablastic neoplasms is where the tumor is effusion alone vs. as solid masses. Clinical and radiological findings are helpful in differentiating PEL from other rare entities. Among plasmablastic neoplasms, Chen et al. proposed a practical diagnostic algorithm using ALK immunostain as the initial screening to identify ALK-positive large B-cell lymphoma [91]. Immunohistochemistry for HHV8-LANA, IgM, kappa and lambda light chains, and EBER may help to differentiate PEL from HHV8-positive DLBCL, NOS (Figure 4).

HHV8-unrelated PEL is usually of B-cell phenotype and generally affects older patients with a history of chronic diseases or fluid overload status. Table 5 lists the major differential diagnoses of HHV8-unrelated type II PEL, including pyothorax-associated lymphoma, DLBCL, NOS, EBV-positive DLBCL, NOS, and HHV8-positive DLBCL, NOS. Pyothorax-associated lymphoma typically affects elderly men with a history of chronic pleural inflammation (tuberculosis or artificial pneumothorax as part of therapy for tuberculosis). Tumor cells are negative for HHV8, but positive for EBV in most cases [89].

In addition to B-cell phenotype, rare HHV8-negative type II PEL cases were reported with plasmablastic, T-cell, or indeterminate phenotype (Supplementary Table S1) [12]. Therefore, Hu et al. suggested that the minimal diagnostic panel for effusion lymphoma with a large cell morphology should include CD3, CD20, PAX5, CD138, IRF4/MUM1, ALK, HHV8, and EBER [9]. However, distinguishing PEL from other lymphomas involving the body cavities is difficult based purely on cytomorphological features [88]. A thorough clinical/imaging evaluation for any possible mass-forming lesions, history of previous or concurrent lymphoma, and underlying medical conditions will prompt additional workup with ancillary tests for a correct diagnosis (Figure 4).

## 9. The Differences of PEL between Western and East Asian Populations

PEL is a rare disease. In the series from the West, the majority of the patients are young to middle-aged HIV-infected patients [19,98]. Baidoun et al. reported 64.6% of HIV-infected individuals and 35.4% of HIV-negative status in a retrospective survey on US cancer database with 178 PEL patients [22]. In contrast, most of the patients reported from East Asia are elderly, without HIV infection [4,9,10,16,38,46,83,88,99]. For example, the Japanese patients were mostly HHV8-negative and older than 65, with only 7.3% cases having HHV8-positive classic PEL [16]. The Korean PEL patients are also elderly, with HHV8-negative type II PEL, and an indolent clinical course [38,46]. Similarly in Taiwan, we previously reported the largest series of PEL and showed a low frequency of association with HHV8 (32%), in contrast to 68% HHV8-negative cases [10].

In an attempt to better understand the geographic differences among patients with HHV8-negative type II PEL, we reviewed 167 cases in the English literature. Interestingly, we found higher rates of underlying medical conditions (68% vs. 31%, *p* < 0.001), HIV infection (20% vs. 2%, *p* = 0.002), iatrogenic immunodeficiency (12% vs. 0%, *p* = 0.003), and EBV co-infection (32% vs. 16%; *p* = 0.042) in the Western population as compared to that from East Asia (Table 3). The relative frequency of immunodeficiency, particularly HIV infection, was low in either Western or East Asian patients. The overall rate of 41.7% (58/139) HHV8-negative type II PEL patients had a fluid overload state, indicating that there are pathogenetic factors other than fluid overload for this disease.

The prognosis of classic PEL is generally poor, with a short survival less than 6 months [8], while the outcome of patients with HHV8-negative type II PEL appears to be more favorable, with a one-year survival rate of 47% among 77 patients (Supplementary Table S1). Alexanian et al. reported that 46.7% of patients survived for at least 1 year after diagnosis [13]. In a Japanese case series by Kaji et al., the majority of patients with HHV8-negative type II PEL responded to systemic treatment, and their prognosis was favorable as compared to that of patients of similar age with classic PEL or DLBCL [16]. However, in Taiwan, the prognosis of patients with both classic and type II PEL were poor [4,10]. More studies of different regions/populations are warranted to elucidate the geographic differences.

## 10. Conclusions

PEL are lymphomatous effusions in body cavities without detectable tumor masses. It is imperative to have a better understanding of this rare neoplasm. Our systemic review shows that HHV8-positive and HHV8-negative PELs exhibit distinctive clinical, cytomorphologic, and immunophenotypic features and they should be considered different variants/subtype of PEL. Diagnoses of PEL remain challenging due to their rarity and considerable overlapping features among HHV8-positive and negative cases, and other nodal or extra-nodal lymphomas. A multidisciplinary approach with incorporation of clinical information, cytomorphology, and various ancillary techniques is the key for correct diagnosis and to distinguish the subtypes of PEL and their mimickers.

## Figures and Tables

**Table 1:** The spectrum of PEL in terms of viral infection and phenotype.

		HIV	HHV8	EBV	Phenotype	References
Type I PEL (HHV8+)	Prototypic PEL by 2017 WHO	+	+	+ (65–80%)	Plasmablastic	[8]
	PEL	-	+	Mostly– (+ in 19–38%)	Plasmablastic	[9,62]
Type II PEL (HHV8−)	EBL by WHO (with fluid overload)	Mostly– (+ in 4%)	-	Mostly– (+ in 29%)	B-cell (majority)	[10,13]
	Without fluid overload	Mostly– (+ in 5%)	-	Mostly– (+ in 29%)	B-cell (majority)	[10,13]

**Table 2:** Comparison of the pertinent clinicopathological features of HHV8-related (classical or type I) and HHV8-unrelated (type II) PEL.

WHO Classification (2017)	Mature B-Cell Neoplasms: Primary Effusion Lymphoma (HHV8-Related)	Effusion-Based Lymphoma (HHV8-Unrelated)	References
Classification by Chen et al. (2018)	Classic or type I PEL	Type II PEL	[10]
HHV8/KSHV	Positive	Negative	[8]
Prevalence	66% of all PEL	33% of all PEL	[10]
Clinical characteristics	Younger patients; HIV infection/immunodeficiency/organ transplantation	Elder patients; HIV-negative/ underlying medical conditions or fluid overload status	
Underlying immune status	Immunocompromised (both HIV-related or HIV-unrelated)	Mostly immunocompetent elder patients with immunosenescence	[9,10,19]
Etiology	HHV8 infection (with possible synergistic effect of EBV)	Fluid overload/chronic inflammatory stimulation/underlying medical conditions (liver cirrhosis, renal dysfunction, or heart failure)	[10,11,12,13,16,38]
EBV association	High (65–80%)	Low (13–30%)	[9,12,13,16,62]
Morphology	Immunoblasts, plasmablastic, anaplastic, or binucleated and multinucleated cells resembling Reed-Sternberg cells	Immunoblastic, plasmablastic, anaplastic, or Burkitt like	
Phenotype	Plasmablastic; Positive for CD45/LCA (90%) and plasma cell-related markers such as CD38, CD138, Blimp-1, and VSc38, but negative for pan B-cell markers	B cell; Positive for CD19, CD20, CD79a, and PAX5, but negative for plasma cell-related markers	
Activation marker (CD30)	Positive (70–100%)	Negative (76–97%)	[9,12,16,44]
*MYC* rearrangement	3%	29%	[10]
Prognosis	Generally poor (median survival <6 months)	Better response to chemotherapy (one-year survival rate 47%)	[8], current study

Abbreviation: PEL, primary effusion lymphoma; EBL, Effusion-based lymphoma; IBL, immunoblastic lymphoma; DLBCL, diffuse large B-cell lymphoma.

**Table 3:** The comparison of main clinicopathological characteristics of HHV8-negative type II PEL patients between Western and Eastern Asian populations.

	Western Populations	East Asian Populations	*p* Value	Total Cases
Fluid overload	55% (22/40)	36% (36/99)	0.068	41.7% (58/139)
Underlying specific medical diseases	68% (28/41)	31% (27/88)	<0.001 *	42.6% (55/129)
Liver cirrhosis	24% (10/41)	8% (7/88)	0.022 *	13.2% (17/129)
Renal dysfunction/ESRD	5% (2/41)	15% (13/88)	0.142	11.6% (15/129)
Heart failure	22% (9/41)	5% (4/88)	0.004 *	10.1% (13/129)
Prior cancer/leukemia	12% (5/41)	3% (3/88)	0.109	6.2% (8/129)
Post-transplantation	5% (2/41)	0% (0/88)	0.099	1.6% (2/129)
**Immunodeficiency Status**				
HIV	20% (6/30)	2% (2/100)	0.002 *	6.2% (8/130)
Iatrogenic immunodeficiency	12% (5/41)	0% (0/88)	0.003 *	3.9% (5/129)
Congenital immunodeficiency	0% (0/41)	1% (1/88)	1.000	0.8% (1/129)
EBV infection	32% (15/47)	16% (15/96)	0.042 *	21.0% (30/143)

* Fisher’s exact test; *p* < 0.05 with statistical significance.

**Table 4:** Differential diagnoses of PEL with a plasmablastic phenotype.

Plasmablastic Lymphoma	Solid tumor, ALK−, EBV+/−, HHV8−, HIV+/−
Plasmablastic myeloma	Clonal bone marrow plasma cells ≥10% or plasmacytoma, CRAB+, ALK−, EBV−, HHV8−, HIV−
ALK-positive LBCL	Solid tumor, ALK+, EBV−, HHV8−, HIV−
HHV8-positive DLBCL, NOS	Solid tumor, ALK−, EBV−, HHV8+, HIV+/−, cytoplasmic IgM with lambda light chain restriction, CD20+/−, CD138−, IRF4/MUM1+

Abbreviations: ALK, anaplastic lymphoma kinase; CRAB, hypercalcemia, renal insufficiency, anemia, and bone lesions; LBCL, large B-cell lymphoma; DLBCL, NOS, diffuse large B-cell lymphoma, not otherwise specified.

**Table 5:** Differential diagnoses of PEL with a B-cell phenotype.

Pyothorax-associated lymphoma	History of pyothorax resulting from artificial pneumothorax for treatment of pulmonary or pleural tuberculosis, ALK−, EBV+, HHV8−, HIV−, CD20+/−, IRF4/MUM1+/−
DLBCL, NOS	Solid tumor, ALK−, EBV−, HHV8−, HIV−, CD20+, CD138−/+
EBV+ DLBCL, NOS	Solid tumor, ALK−, EBV+, HHV8−, HIV−, CD20+/−, IRF4/MUM1+
HHV8-positive DLBCL, NOS	Solid tumor, ALK−, EBV−, HHV8+, HIV+/−, cytoplasmic IgM with lambda light chain restriction, CD20+/−, CD138−, IRF4/MUM1+

## Data Availability

Data are available on request due to all institutional restrictions related to patient privacy.

## Supplementary Materials

The following supporting information can be downloaded at: https://www.mdpi.com/article/10.3390/diagnostics12030713/s1, Table S1: Literature review of HHV8-negative or type II PEL cases.

## References

[1] Das D.K. (2006). Serous effusions in malignant lymphomas: A review. Diagn. Cytopathol..

[2] Dunphy C.H. (1996). Combined cytomorphologic and immunophenotypic approach to evaluation of effusions for lymphomatous involvement. Diagn. Cytopathol..

[3] Das D.K., Al-Juwaiser A., George S.S., Francis I.M., Sathar S.S., Sheikh Z.A., Shaheen A., Pathan S.K., Haji B.E., George J. (2007). Cytomorphological and immunocytochemical study of non-Hodgkin’s lymphoma in pleural effusion and ascitic fluid. Cytopathology.

[4] Wang R.C., Chen Y.H., Chen B.J., Chuang S.S. (2021). The cytopathological spectrum of lymphomas in effusions in a tertiary center in Taiwan. Diagn. Cytopathol..

[5] Pereira T.C., Saad R.S., Liu Y., Silverman J.F. (2006). The diagnosis of malignancy in effusion cytology: A pattern recognition approach. Adv. Anat. Pathol..

[6] Knowles D.M., Inghirami G., Ubriaco A., Dalla-Favera R. (1989). Molecular genetic analysis of three AIDS-associated neoplasms of uncertain lineage demonstrates their B-cell derivation and the possible pathogenetic role of the Epstein-Barr virus. Blood.

[7] Nador R.G., Cesarman E., Chadburn A., Dawson D.B., Ansari M.Q., Sald J., Knowles D.M. (1996). Primary effusion lymphoma: A distinct clinicopathologic entity associated with the Kaposi’s sarcoma-associated herpes virus. Blood.

[8] Said J., Cesarman E. (2017). Primary effusion lymphoma. WHO Classification of Tumours of Haematopoietic and Lymphoid Tissues.

[9] Hu Z., Pan Z., Chen W., Shi Y., Wang W., Yuan J., Wang E., Zhang S., Kurt H., Mai B. (2021). Primary Effusion Lymphoma: A Clinicopathological Study of 70 Cases. Cancers.

[10] Chen B.J., Wang R.C., Ho C.H., Yuan C.T., Huang W.T., Yang S.F., Hsieh P.P., Yung Y.C., Lin S.Y., Hsu C.F. (2018). Primary effusion lymphoma in Taiwan shows two distinctive clinicopathological subtypes with rare human immunodeficiency virus association. Histopathology.

[11] Xiao J., Selvaggi S.M., Leith C.P., Fitzgerald S.A., Stewart J. (2013). Kaposi sarcoma herpesvirus/human herpesvirus-8-negative effusion-based lymphoma: Report of 3 cases and review of the literature. Cancer Cytopathol..

[12] Wu W., Youm W., Rezk S.A., Zhao X. (2013). Human herpesvirus 8-unrelated primary effusion lymphoma-like lymphoma: Report of a rare case and review of 54 cases in the literature. Am. J. Clin. Pathol..

[13] Alexanian S., Said J., Lones M., Pullarkat S.T. (2013). KSHV/HHV8-negative effusion-based lymphoma, a distinct entity associated with fluid overload states. Am. J. Surg. Pathol..

[14] Usmani A., Walts A.E., Patel S., Alkan S., Kitahara S. (2015). HHV8-negative effusion based lymphoma: A series of 17 cases at a single institution. J. Am. Soc. Cytopathol..

[15] Chen B.J., Chen D.Y., Kuo C.C., Chuang S.S. (2017). EBV-associated but HHV8-unrelated double-hit effusion-based lymphoma. Diagn. Cytopathol..

[16] Kaji D., Ota Y., Sato Y., Nagafuji K., Ueda Y., Okamoto M., Terasaki Y., Tsuyama N., Matsue K., Kinoshita T. (2020). Primary human herpesvirus 8-negative effusion-based lymphoma: A large B-cell lymphoma with favorable prognosis. Blood. Adv..

[17] Fiori S., Todisco E., Ramadan S., Gigli F., Falco P., Iurlo A., Rampinelli C., Croci G., Pileri S.A., Tarella C. (2021). HHV8-Negative Effusion-Based Large B Cell Lymphoma Arising in Chronic Myeloid Leukemia Patients under Dasatinib Treatment: A Report of Two Cases. Biology.

[18] Carbone A., Gloghini A. (2008). PEL and HHV8-unrelated effusion lymphomas: Classification and diagnosis. Cancer.

[19] Rossi G., Cozzi I., Della Starza I., De Novi L.A., De Propris M.S., Gaeta A., Petrucci L., Pulsoni A., Pulvirenti F., Ascoli V. (2021). Human herpesvirus-8-positive primary effusion lymphoma in HIV-negative patients: Single institution case series with a multidisciplinary characterization. Cancer Cytopathol..

[20] Yuan L., Cook J.R., Elsheikh T.M. (2020). Primary effusion lymphoma in human immune deficiency (HIV)-negative non-organ transplant immunocompetent patients. Diagn. Cytopathol..

[21] Nicola M., Onorati M., Bianchi C.L., Pepe G., Bellone S., Di Nuovo F. (2015). Primary Effusion Lymphoma: Cytological Diagnosis of a Rare Entity--Report of Two Cases in HIV-Uninfected Patients from a Single Institution. Acta Cytol..

[22] Baidoun F., Moustafa M.A., Tun H.W., Hill B.T. (2022). Clinical Characteristics and Survival Outcomes of Primary Effusion Lymphoma: A National Cancer Database Study. Clin. Lymphoma Myeloma Leuk..

[23] Swerdlow S.H., Campo E., Harris N.L., Jaffe E.S., Pileri S., Stein H., Thiele J., Organizacja Z.S., International Agency for Research on Cancer (2017). WHO Classification of Tumours of Haematopoietic and Lymphoid Tissues.

[24] Shimada K., Hayakawa F., Kiyoi H. (2018). Biology and management of primary effusion lymphoma. Blood.

[25] Karcher D.S. (2021). From HIV to Coronavirus Disease 2019 (COVID-19): Hematologic Complications in Viral Pandemics. Arch. Pathol. Lab. Med..

[26] Fajgenbaum D.C., June C.H. (2020). Cytokine Storm. N. Engl. J. Med..

[27] Mehta P., Fajgenbaum D.C. (2021). Is severe COVID-19 a cytokine storm syndrome: A hyperinflammatory debate. Curr. Opin. Rheumatol..

[28] Zanza C., Romenskaya T., Manetti A.C., Franceschi F., La Russa R., Bertozzi G., Maiese A., Savioli G., Volonnino G., Longhitano Y. (2022). Cytokine Storm in COVID-19: Immunopathogenesis and Therapy. Medicina.

[29] Dolcetti R., Gloghini A., Caruso A., Carbone A. (2016). A lymphomagenic role for HIV beyond immune suppression?. Blood.

[30] Momose S., Tamaru J.I. (2019). Iatrogenic immunodeficiency-associated lymphoproliferative disorders of B-cell type that develop in patients receiving immunosuppressive drugs other than in the post-transplant setting. J. Clin. Exp. Hematop..

[31] Swerdlow S.H., Webber S.A., Chadburn A., Ferry J.A. (2017). Post-transplant lymphoproliferative disorders. WHO Classification of Tumours of Haematopoietic and Lymphoid Tissues.

[32] Jones D., Ballestas M.E., Kaye K.M., Gulizia J.M., Winters G.L., Fletcher J., Scadden D.T., Aster J.C. (1998). Primary-effusion lymphoma and Kaposi’s sarcoma in a cardiac-transplant recipient. N. Engl. J. Med..

[33] Zanelli M., Sanguedolce F., Zizzo M., Palicelli A., Bassi M.C., Santandrea G., Martino G., Soriano A., Caprera C., Corsi M. (2021). Primary effusion lymphoma occurring in the setting of transplanted patients: A systematic review of a rare, life-threatening post-transplantation occurrence. BMC Cancer.

[34] Boulanger E., Afonso P.V., Yahiaoui Y., Adle-Biassette H., Gabarre J., Agbalika F. (2008). Human herpesvirus-8 (HHV-8)-associated primary effusion lymphoma in two renal transplant recipients receiving rapamycin. Am. J. Transplant..

[35] Riva G., Luppi M., Barozzi P., Forghieri F., Potenza L. (2012). How I treat HHV8/KSHV-related diseases in posttransplant patients. Blood.

[36] Perier A., Savey L., Marcelin A.G., Serve P., Saadoun D., Barete S. (2017). De Novo Human Herpesvirus 8 Tumors Induced by Rituximab in Autoimmune or Inflammatory Systemic Diseases. Arthritis Rheumatol..

[37] Okada K., Asakura S., Yano T., Kishimoto T. (2018). EBV-positive PEL-like lymphoma that developed in the course of antisynthetase syndrome treated with tacrolimus. Int. J. Hematol..

[38] Kim M., An J., Yoon S.O., Yong S.H., Kim J.S., Yang W.I., Leem A.Y. (2020). Human herpesvirus 8-negative effusion-based lymphoma with indolent clinical behavior in an elderly patient: A case report and literature review. Oncol. Lett..

[39] Mancuso S., Carlisi M., Santoro M., Napolitano M., Raso S., Siragusa S. (2018). Immunosenescence and lymphomagenesis. Immun. Ageing..

[40] Sarkozy C., Salles G., Falandry C. (2015). The biology of aging and lymphoma: A complex interplay. Curr. Oncol. Rep..

[41] Zanelli M., Zizzo M., Bisagni A., Froio E., De Marco L., Valli R., Filosa A., Luminari S., Martino G., Massaro F. (2020). Germinotropic lymphoproliferative disorder: A systematic review. Ann. Hematol..

[42] Gonzalez-Farre B., Martinez D., Lopez-Guerra M., Xipell M., Monclus E., Rovira J., Garcia F., Lopez-Guillermo A., Colomo L., Campo E. (2017). HHV8-related lymphoid proliferations: A broad spectrum of lesions from reactive lymphoid hyperplasia to overt lymphoma. Mod. Pathol..

[43] Verga L., Leni D., Cazzaniga G., Crosta S., Seminati D., Rossi M., L’Imperio V., Pagni F. (2020). The spectrum of the cytopathological features of primary effusion lymphoma and human herpes virus 8-related lymphoproliferative disorders. Cytopathology.

[44] Carbone A., Gloghini A. (2008). KSHV/HHV8-associated lymphomas. Br. J. Haematol..

[45] Vega F., Miranda R.N., Medeiros L.J. (2020). KSHV/HHV8-positive large B-cell lymphomas and associated diseases: A heterogeneous group of lymphoproliferative processes with significant clinicopathological overlap. Mod. Pathol..

[46] Shin J., Lee J.O., Choe J.Y., Bang S.M., Lee J.S. (2017). Human Herpesvirus 8-Unrelated Primary Effusion Lymphoma-Like Lymphoma in an Elderly Korean Patient with a Good Response to Rituximab Plus Cyclophosphamide, Doxorubicin, Vincristine, and Prednisolone. Cancer Res. Treat..

[47] Choi J.W., Kim Y., Lee J.H., Kim Y.S. (2015). Human Herpesvirus 8-Negative and Epstein-Barr Virus-Positive Effusion-Based Lymphoma in a Patient with Human Immunodeficiency Virus. J. Pathol. Transl. Med..

[48] Mohammad F., Siddique M.N., Siddiqui F., Popalzai M., Asgari M., Odaimi M. (2014). A Unique Case of Malignant Pleuropericardial Effusion: HHV-8-Unrelated PEL-Like Lymphoma-A Case Report and Review of the Literature. Case Rep. Oncol. Med..

[49] Fan H.B., Yang D.L., Guo Y., Chen A.S., Zhou M.X., Wu J.J., Ma X.J., Li Z. (2014). Human herpes virus 8-unrelated primary effusion lymphoma-like lymphoma in a patient with hepatitis B virus-related liver cirrhosis: A case report. J. Res. Med. Sci..

[50] Sumida K., Ubara Y., Takaichi K., Wake A. (2012). Primary effusion lymphoma-like lymphoma with polycystic kidney disease. BMJ Case Rep..

[51] Kim K.H., Lee J.H., Jeong H.C., Kim G.W., Song S.H., Jung S.Y., Kim G.I., Kim E.K. (2012). A case of human herpes virus-8 unrelated primary effusion lymphoma-like lymphoma presented as pleural effusion. Tuberc. Respir. Dis..

[52] Wang T., Nava V.E., Schechter G.P., Lichy J.H., Liu M.L. (2011). Human herpes virus 8-unrelated primary effusion lymphoma-like lymphoma: A patient successfully treated with pleurodesis. J. Clin. Oncol..

[53] Terasaki Y., Yamamoto H., Kiyokawa H., Okumura H., Saito K., Ichinohasama R., Ishida Y. (2011). Disappearance of malignant cells by effusion drainage alone in two patients with HHV-8-unrelated HIV-negative primary effusion lymphoma-like lymphoma. Int. J. Hematol..

[54] Kagoya Y., Takahashi T., Yoshimoto T., Ichikawa M., Hangaishi A., Fukayama M., Kurokawa M. (2011). Recurrent pericardial effusion after treatment for primary effusion lymphoma-like lymphoma: An autopsied case. Ann. Hematol..

[55] Takahashi T., Hangaishi A., Yamamoto G., Ichikawa M., Imai Y., Kurokawa M. (2010). HIV-negative, HHV-8-unrelated primary effusion lymphoma-like lymphoma: Report of two cases. Am. J. Hematol..

[56] Cooper A.R., Burack W.R., Allerton J.P. (2010). A case of Kaposi sarcoma-associated herpesvirus/human herpesvirus 8-unrelated but Epstein-Barr virus-positive primary effusion lymphoma-like lymphoma in the setting of human immunodeficiency virus and hepatitis C virus infection. Leuk. Lymphoma.

[57] Tsagarakis N.J., Argyrou A., Gortzolidis G., Kentrou N., Papadhimitriou S.I., Tzanetou K., Kakiopoulos G., Papadimitriou K.A., Skoumi D., Paterakis G. (2009). Report of an HIV and HHV-8 negative case of primary effusion lymphoma with idiopathic T4 lymphocytopenia. Int. J. Hematol..

[58] De Filippi R., Iaccarino G., Frigeri F., Di Francia R., Crisci S., Capobianco G., Arcamone M., Becchimanzi C., Amoroso B., De Chiara A. (2009). Elevation of clonal serum free light chains in patients with HIV-negative primary effusion lymphoma (PEL) and PEL-like lymphoma. Br. J. Haematol..

[59] Adiguzel C., Bozkurt S.U., Kaygusuz I., Uzay A., Tecimer T., Bayik M. (2009). Human herpes virus 8-unrelated primary effusion lymphoma-like lymphoma: Report of a rare case and review of the literature. APMIS.

[60] Terasaki Y., Okumura H., Saito K., Sato Y., Yoshino T., Ichinohasama R., Ishida Y. (2008). HHV-8/KSHV-negative and CD20-positive primary effusion lymphoma successfully treated by pleural drainage followed by chemotherapy containing rituximab. Intern. Med..

[61] Niino D., Tsukasaki K., Torii K., Imanishi D., Tsuchiya T., Onimaru Y., Tsushima H., Yoshida S., Yamada Y., Kamihira S. (2008). Human herpes virus 8-negative primary effusion lymphoma with BCL6 rearrangement in a patient with idiopathic CD4 positive T-lymphocytopenia. Haematologica.

[62] Kobayashi Y., Kamitsuji Y., Kuroda J., Tsunoda S., Uoshima N., Kimura S., Wada K., Matsumoto Y., Nomura K., Horiike S. (2007). Comparison of human herpes virus 8 related primary effusion lymphoma with human herpes virus 8 unrelated primary effusion lymphoma-like lymphoma on the basis of HIV: Report of 2 cases and review of 212 cases in the literature. Acta Haematol..

[63] Youngster I., Vaisben E., Cohen H., Nassar F. (2006). An unusual cause of pleural effusion. Age Ageing.

[64] Venizelos I., Tamiolakis D., Lambropoulou M., Nikolaidou S., Bolioti S., Papadopoulos H., Papadopoulos N. (2005). An unusual case of posttransplant peritoneal primary effusion lymphoma with T-cell phenotype in a HIV-negative female, not associated with HHV-8. Pathol. Oncol. Res..

[65] Matsumoto Y., Nomura K., Ueda K., Satoh K., Yasuda N., Taki T., Yokota S., Horiike S., Okanoue T., Taniwaki M. (2005). Human herpesvirus 8-negative malignant effusion lymphoma: A distinct clinical entity and successful treatment with rituximab. Leuk. Lymphoma.

[66] Jenkins C., Sorour Y., Blake E., Elliot R., Al-Sabah A.I., Green J. (2005). Human-immunodeficiency-virus-negative, human-herpes-virus-8-negative abdominal cavity primary effusion lymphoma. Clin. Oncol..

[67] Fujiwara T., Ichinohasama R., Miura I., Sugawara T., Harigae H., Yokoyama H., Takahashi S., Tomiya Y., Yamada M., Ishizawa K. (2005). Primary effusion lymphoma of the pericardial cavity carrying t(1;22)(q21;q11) and t(14;17)(q32;q23). Cancer Genet. Cytogenet..

[68] Takao T., Kobayashi Y., Kuroda J., Omoto A., Nishimura T., Kamitsuji Y., Fukiya E., Nakamura C., Kimura S., Yoshikawa T. (2004). Rituximab is effective for human herpesvirus-8-negative primary effusion lymphoma with CD20 phenotype associated hepatitis C virus-related liver cirrhosis. Am. J. Hematol..

[69] Nonami A., Yokoyama T., Takeshita M., Ohshima K., Kubota A., Okamura S. (2004). Human herpes virus 8-negative primary effusion lymphoma (PEL) in a patient after repeated chylous ascites and chylothorax. Intern. Med..

[70] Inoue Y., Tsukasaki K., Nagai K., Soda H., Tomonaga M. (2004). Durable remission by sobuzoxane in an HIV-seronegative patient with human herpesvirus 8-negative primary effusion lymphoma. Int. J. Hematol..

[71] Shimazaki M., Fujita M., Tsukamoto K., Matsuki T., Iwata M., Takahashi H., Doi A., Hyakkoku M., Yamauchi K., Genda S. (2003). An unusual case of primary effusion lymphoma in a HIV-negative patient not pathogenetically associated with HHV8. Eur. J. Haematol..

[72] Paner G.P., Jensen J., Foreman K.E., Reyes C.V. (2003). HIV and HHV-8 negative primary effusion lymphoma in a patient with hepatitis C virus-related liver cirrhosis. Leuk. Lymphoma.

[73] Nakamura Y., Tajima F., Omura H., Ishiga K., Kawatani T., Murawaki Y. (2003). Primary effusion lymphoma of the left scrotum. Intern. Med..

[74] Hisamoto A., Yamane H., Hiraki A., Maeda Y., Fujii N., Sasaki K., Miyake T., Sasaki T., Nakamura T., Kiura K. (2003). Human herpes virus-8-negative primary effusion lymphoma in a patient with common variable immunodeficiency. Leuk. Lymphoma.

[75] Chiba H., Matsunaga T., Kuribayashi K., Nikaido T., Shirao S., Murakami K., Hirayama Y., Sakamaki S., Ikeda H., Niitsu Y. (2003). Autoimmune hemolytic anemia as a first manifestation of primary effusion lymphoma. Ann. Hematol..

[76] Ohshima K., Ishiguro M., Yamasaki S., Miyagi J., Okamura S., Sugio Y., Muta T., Sasaki H., Tuchiya T., Kawasaki C. (2002). Chromosomal and comparative genomic analyses of HHV-8-negative primary effusion lymphoma in five HIV-negative Japanese patients. Leuk. Lymphoma.

[77] Yamamoto Y., Kitajima H., Sakihana H., Shigeki T., Fukuhara S. (2001). CD3+CD4-CD8-TCR-alphabeta+ T-cell lymphoma with clinical features of primary effusion lymphoma: An autopsy case. Int. J. Hematol..

[78] Tanaka S., Katano H., Tsukamoto K., Jin M., Oikawa S., Nishihara H., Sawa H., Sawada K., Shimizu M., Sata T. (2001). HHV8-negative primary effusion lymphoma of the peritoneal cavity presenting with a distinct immunohistochemical phenotype. Pathol. Int..

[79] Rodriguez J., Romaguera J.E., Katz R.L., Said J., Cabanillas F. (2001). Primary effusion lymphoma in an HIV-negative patient with no serologic evidence of Kaposi’s sarcoma virus. Leuk. Lymphoma..

[80] Ohori N.P., Whisnant R.E., Nalesnik M.A., Swerdlow S.H. (2001). Primary pleural effusion posttransplant lymphoproliferative disorder: Distinction from secondary involvement and effusion lymphoma. Diagn. Cytopathol..

[81] Hara T., Nishi S., Horimoto A., Takenaka S., Ibata Y., Akamatsu H. (2001). Primary effusion lymphoma in a patient with hepatitis C virus-related liver cirrhosis. J. Gastroenterol. Hepatol..

[82] Ashihara E., Shimazaki C., Hirai H., Inaba T., Hasegawa G., Mori S., Nakagawa M. (2001). Human herpes virus 8-negative primary effusion lymphoma in a patient with a ventriculoperitoneal shunt tube. Int. J. Hematol..

[83] Ichinohasama R., Miura I., Kobayashi N., Saitoh Y., DeCoteau J.F., Saiki Y., Mori S., Kadin M.E., Ooya K. (1998). Herpes virus type 8-negative primary effusion lymphoma associated with PAX-5 gene rearrangement and hepatitis C virus: A case report and review of the literature. Am. J. Surg. Pathol..

[84] Carbone A., Cilia A.M., Gloghini A., Canzonieri V., Pastore C., Todesco M., Cozzi M., Perin T., Volpe R., Pinto A. (1997). Establishment of HHV-8-positive and HHV-8-negative lymphoma cell lines from primary lymphomatous effusions. Int. J. Cancer.

[85] Ascoli V., Lo Coco F., Artini M., Levrero M., Fruscalzo A., Mecucci C. (1997). Primary effusion Burkitt’s lymphoma with t(8;22) in a patient with hepatitis C virus-related cirrhosis. Hum. Pathol..

[86] Hermine O., Michel M., Buzyn-Veil A., Gessain A. (1996). Body-cavity-based lymphoma in an HIV-seronegative patient without Kaposi’s sarcoma-associated herpesvirus-like DNA sequences. N. Engl. J. Med..

[87] Carbone A., Gloghini A., Vaccher E., Zagonel V., Pastore C., Dalla Palma P., Branz F., Saglio G., Volpe R., Tirelli U. (1996). Kaposi’s sarcoma-associated herpesvirus DNA sequences in AIDS-related and AIDS-unrelated lymphomatous effusions. Br. J. Haematol..

[88] Kishimoto K., Kitamura T., Hirayama Y., Tate G., Mitsuya T. (2009). Cytologic and immunocytochemical features of EBV negative primary effusion lymphoma: Report on seven Japanese cases. Diagn. Cytopathol..

[89] Kim Y., Park C.J., Roh J., Huh J. (2014). Current concepts in primary effusion lymphoma and other effusion-based lymphomas. Korean J. Pathol..

[90] Wang W., Kanagal-Shamanna R., Medeiros L.J. (2018). Lymphoproliferative disorders with concurrent HHV8 and EBV infection: Beyond primary effusion lymphoma and germinotropic lymphoproliferative disorder. Histopathology.

[91] Chen B.J., Chuang S.S. (2020). Lymphoid Neoplasms with Plasmablastic Differentiation: A Comprehensive Review and Diagnostic Approaches. Adv. Anat. Pathol..

[92] Chen Y.B., Rahemtullah A., Hochberg E. (2007). Primary effusion lymphoma. Oncologist.

[93] Pan Z.G., Zhang Q.Y., Lu Z.B., Quinto T., Rozenvald I.B., Liu L.T., Wilson D., Reddy V., Huang Q., Wang H.Y. (2012). Extracavitary KSHV-associated large B-Cell lymphoma: A distinct entity or a subtype of primary effusion lymphoma? Study of 9 cases and review of an additional 43 cases. Am. J. Surg. Pathol..

[94] Banks P.M., Warnke R.A. (2001). Primary effusion lymphoma. WHO Classification of Tumours of Haematopoietic and Lymphoid Tissues.

[95] Liu C.Y., Chuang S.S. (2021). A Simple and Practical Guide for Triaging Lymphocyte-rich Effusions for Ancillary Studies. Adv. Anat. Pathol..

[96] Calvani J., Gerard L., Fadlallah J., Poullot E., Galicier L., Robe C., Garzaro M., Bertinchamp R., Boutboul D., Cuccuini W. (2021). A Comprehensive Clinicopathologic and Molecular Study of 19 Primary Effusion Lymphomas in HIV-infected Patients. Am. J. Surg. Pathol..

[97] Masir N., Marafioti T., Jones M., Natkunam Y., Rudiger T., Hansmann M.L., Mason D.Y. (2006). Loss of CD19 expression in B-cell neoplasms. Histopathology.

[98] El-Fattah M.A. (2017). Clinical characteristics and survival outcome of primary effusion lymphoma: A review of 105 patients. Hematol. Oncol..

[99] Sasaki Y., Isegawa T., Shimabukuro A., Yonaha T., Yonaha H. (2014). Primary Effusion Lymphoma in an Elderly HIV-Negative Patient with Hemodialysis: Importance of Evaluation for Pleural Effusion in Patients Receiving Hemodialysis. Case Rep. Nephrol. Urol..

