# Supplementary material for: Primary Effusion Lymphoma: A Timely Review on the Association with HIV, HHV8, and EBV
Source: Diagnostics (Basel). 2022 Mar 15;12(3):713. doi: 10.3390/diagnostics12030713 (PMC8947353; doi:10.3390/diagnostics12030713)
Supplement: Supplementary file 1 [file diagnostics-12-00713-s001.zip › diagnostics-1620664-supplementary.pdf]

Table S1. Literature review of HHV8-negative or type II PEL cases.

Authors (year)	Case No.	Associated Medical Conditions	HIV Positive	HBV Positive	HCV Positive	Fluid Overload	EBV Positive	*MYC* Rearrangement	Phenotype/ Genotype	Median Survival (months)	1-year Survival Rate
Nador et al. (1996) [1]	4	NA	100% (4/4)	NA	NA	NA	100% (4/4)	100% (4/4)	Indeterminate	6	0% (0/3)
Hermine et al. (1996) [2]	1	NA	0% (0/1)	NA	NA	NA	0% (0/1)	NA	B-cell	NA	NA
Carbone et al. (1996) [3]	2	NA	50% (1/2)	NA	NA	NA	50% (1/2)	NA	B-cell	3	0% (0/2)
Ascoli et al. (1997) [4]	1	LC	0% (0/1)	NA	100% (1/1)	100% (1/1)	NA	100% (1/1)	B-cell	2	0% (0/1)
Carbone et al. (1997) [5]	1	NA	0% (0/1)	NA	NA	NA	100% (1/1)	100% (1/1)	B-cell	0.3	0% (0/1)
Ichinohasama et al. (1998) [6]	1	LC	0% (0/1)	NA	100% (1/1)	100% (1/1)	0% (0/1)	0% (0/1)	B-cell	10	0% (0/1)
Tanaka et al. (2001) [7]	1	No	0% (0/1)	NA	0% (0/1)	NA	0% (0/1)	0% (0/1)	B-cell	3	0% (0/1)
Ashihara et al. (2001) [8]	1	Brain tumor	0% (0/1)	0% (0/1)	0% (0/1)	100% (1/1)	100% (1/1)	NA	B-cell	24	100% (1/1)
Hara et al. (2001) [9]	1	LC	0% (0/1)	0% (0/1)	100% (1/1)	100% (1/1)	NA	NA	B-cell	NA	NA
Ohori et al. (2001) [10]	1	LC; liver transplantation	NA	100% (1/1)	NA	0% (0/1)	100% (1/1)	NA	B-cell	NA	NA
Rodriguez et al. (2001) [11]	1	LC	0% (0/1)	NA	NA	100% (1/1)	100% (1/1)	NA	B-cell	NA	NA
Yamamoto et al. (2001) [12]	1	cerebral aneurysm	0% (0/1)	0% (0/1)	0% (0/1)	0% (0/1)	0% (0/1)	NA	T-cell	5	0% (0/1)
Ohshima et al. (2002) [13]	5	NA	0% (0/5)	NA	NA	NA	20% (1/5)	80% (4/5)	B-cell	NA	NA
Hisamoto et al. (2003) [14]	1	common variable immunodeficiency	0% (0/1)	NA	NA	100% (1/1)	100% (1/1)	NA	B-cell	0.6	0% (0/1)
Chiba et al. (2003) [15]	1	autoimmune hemolytic anemia	0% (0/1)	0% (0/1)	0% (0/1)	0% (0/1)	100% (1/1)	0% (0/1)	B-cell	3	0% (0/1)
Nakamura et al. (2003) [16]	1	left scrotum swelling	0% (0/1)	0% (0/1)	0% (0/1)	0% (0/1)	0% (0/1)	NA	B-cell	NA	NA
Paner et al. (2003) [17]	1	LC	0% (0/1)	NA	100% (1/1)	100% (1/1)	0% (0/1)	NA	B-cell	5	0% (0/1)
Shimazaki et al. (2003) [18]	1	AF	0% (0/1)	0% (0/1)	0% (0/1)	100% (1/1)	0% (0/1)	100% (1/1)	B-cell	5	0% (0/1)
Inoue et al. (2004) [19]	1	HF	0% (0/1)	0% (0/1)	0% (0/1)	100% (1/1)	0% (0/1)	0% (0/1)	B-cell	18	100% (1/1)
Nonami et al. (2004) [20]	1	chylothorax	0% (0/1)	0% (0/1)	100% (1/1)	100% (1/1)	0% (0/1)	100% (1/1)	B-cell	22	100% (1/1)
Takao et al. (2004) [21]	1	LC	0% (0/1)	0% (0/1)	100% (1/1)	100% (1/1)	0% (0/1)	100% (1/1)	B-cell	28	100% (1/1)
Fujiwara et al. (2005) [22]	1	No	0% (0/1)	NA	0% (0/1)	NA	0% (0/1)	0% (0/1)	B-cell	36	100% (1/1)
Jenkins et al. (2005) [23]	1	LC	0% (0/1)	NA	NA	100% (1/1)	NA	NA	Plasmablastic	NA	NA

Matsumoto et al. (2005) [24]	2	No	0% (0/2)	NA	NA	NA	0% (0/2)	NA	B-cell	NA	NA
Venizelos et al. (2005) [25]	1	renal transplantation	0% (0/1)	NA	NA	100% (1/1)	0% (0/1)	NA	T cell	1	0% (0/1)
Youngster et al. (2006) [26]	1	HF	0% (0/1)	0% (0/1)	0% (0/1)	100% (1/1)	0% (0/1)	NA	B-cell	NA	NA
Kobayashi et al. (2007) [27]	2	LC	0% (0/2)	NA	100% (1/1)	100% (1/1)	50% (1/2)	NA	B-cell	22	100% (2/2)
Niino et al. (2008) [28]	1	HF	0% (0/1)	0% (0/1)	0% (0/1)	100% (1/1)	100% (1/1)	NA	B-cell	30	100% (1/1)
Terasaki et al. (2008) [29]	1	No	0% (0/1)	NA	0% (0/1)	0% (0/1)	0% (0/1)	0% (0/1)	B-cell	22	100% (1/1)
Adiguzel et al. (2009) [30]	1	HTN ; CAD	0% (0/1)	NA	0% (0/1)	100% (1/1)	0% (0/1)	NA	Indeterminate	40	100% (1/1)
De Filippi et al. (2009) [31]	1	LC	0% (0/1)	NA	100% (1/1)	0% (0/1)	100% (1/1)	NA	Plasmablastic	9	0% (0/1)
Tsagarakis et al. (2009) [32]	1	AMI	0% (0/1)	100% (1/1)	0% (0/1)	100% (1/1)	100% (1/1)	0% (0/1)	B-cell	NA	NA
Takahashi et al. (2010) [33]	2	NA	0% (0/2)	NA	NA	NA	50% (1/2)	NA	B-cell	12	100% (1/1)
Cooper et al. (2010) [34]	1	HIV immunocompromised	100% (1/1)	0% (0/1)	100% (1/1)	NA	100% (1/1)	100% (1/1)	B-cell	2	0% (0/1)
Kagoya et al. (2011) [35]	1	HF	0% (0/1)	NA	0% (0/1)	100% (1/1)	0% (0/1)	NA	B-cell	3	0% (0/1)
Terasaki et al. (2011) [36]	2	HF	0% (0/2)	NA	0% (0/2)	100% (2/2)	0% (0/2)	50% (1/2)	B-cell	14	100% (2/2)
Wang et al. (2011) [37]	1	HTN	0% (0/1)	0% (0/1)	0% (0/1)	100% (1/1)	0% (0/1)	NA	B-cell	55	100% (1/1)
Sumida et al. (2012) [38]	1	polycystic kidney disease	0% (0/1)	NA	NA	100% (1/1)	NA	NA	B-cell	18	100% (1/1)
Kim et al. (2012) [39]	1	HTN	0% (0/1)	NA	0% (0/1)	0% (0/1)	0% (0/1)	NA	B-cell	18	100% (1/1)
Alexanian et al. (2013) [40]	5	CAD (2), LC (2), HF (1)	0% (0/2)	NA	33% (1/3)	60% (3/5)	20% (1/5)	NA	B-cell	2	0% (0/3)
Wu et al. (2013) [41]	1	No	0% (0/1)	0% (0/1)	0% (0/1)	0% (0/1)	0% (0/1)	0% (0/1)	B-cell	5.5	0% (0/1)
Xiao et al. (2013) [42]	3	LC (1), AF (1), pancreatitis (1)	0% (0/1)	0% (0/1)	0% (0/1)	33% (1/3)	0% (0/3)	100% (1/1)	B-cell 67% (2/3); Indeterminate 33% (1/3)	25.3	66.7% (2/3)
Mohammad et al. (2014) [43]	1	HTN, AF	0% (0/1)	NA	0% (0/1)	0% (0/1)	0% (0/1)	NA	B-cell	14	100% (1/1)
Fan et al. (2014) [44]	1	LC	0% (0/1)	100% (1/1)	0% (0/1)	0% (0/1)	0% (0/1)	NA	B-cell	0.3	0% (0/1)
Choi et al. (2015) [45]	1	HIV immunocompromised	100% (1/1)	0% (0/1)	0% (0/1)	0% (0/1)	100% (1/1)	NA	B-cell	NA	NA

Usmani et al. (2015) [46]	17	HF (7), ESRD (1), Breast ca (2), CAD (1), LBBB (1), HTN (1), COPD (1), PE (1), FL (1), renal disease (1)	0% (0/4)	NA	16.7% (1/6)	47.1% (8/17)	5.9% (1/17)	21.4% (3/14)	B-cell 88% (15/17); Indeterminate 12% (2/17)	47.3	45.5% (5/11)
Shin et al. (2017) [47]	1	Pitting edema	0% (0/1)	0% (0/1)	0% (0/1)	100% (1/1)	0% (0/1)	NA	B-cell	15	100% (1/1)
Chen et al. (2018) [48]	17	Prior cancers (3), CRF (2)	14% (1/7)	0% (0/7)	10% (1/10)	50% (7/14)	13% (2/16)	27% (3/11)	B-cell 77% (13/17); Plasmablastic 18% (3/17); Indeterminate 6% (1/17)	2.7	31% (5/16)
Kaji et al. (2020) [49]	64	Arrhythmia (27), renal dysfunction(11), myocardial infarction (10), hypothyroidism (8), LC (3)	0% (0/58)	0% (0/62)	1.6% (1/61)	20% (12/60)	13.3% (6/45)	19.4% (7/36)	B-cell 100% (64/64)	Median survival time after relapse: 13 months	2-year OS rate: 84.7%
Kim et al. (2020) [50]	1	HTN	0% (0/1)	0% (0/1)	0% (0/1)	0% (0/1)	0% (0/1)	0% (0/1)	B-cell	22	100% (1/1)
Fiori et al. (2021) [51]	2	CML (2)	NA	NA	NA	50% (1/2)	50% (1/2)	50% (1/2)	B-cell 100% (2/2)	16.5	100% (2/2)
Wang RC et al. (2021) [52]	2	No	0% (0/2)	NA	0% (0/2)	100% (2/2)	0% (0/2)	NA	B-cell 100% (2/2)	13	100% (2/2)
Subtotal	167	LC 13% (17/129), renal dysfunction 12% (15/129), HF 10% (13/129), prior cancer/leukemia 6% (8/129), post-transplantation 2% (2/129)	6% (8/130)	3% (3/90)	12% (14/116)	42% (58/139)	21% (30/143)	34% (31/90)	B-cell 92% (154/167); Plasmablastic 3% (5/167); T-cell 1% (2/167); indeterminate 4% (6/167)	–	47% (36/77)

References

1. Nador, R.G.; Cesarman, E.; Chadburn, A.; Dawson, D.B.; Ansari, M.Q.; Sald, J.; Knowles, D.M. Primary effusion lymphoma: A distinct clinicopathologic entity associated with the Kaposi's sarcoma-associated herpes virus. *Blood* **1996**, *88*, 645–656.
2. Hermine, O.; Michel, M.; Buzyn-Veil, A.; Gessain, A. Body-cavity-based lymphoma in an HIV-seronegative patient without Kaposi's sarcoma-associated herpesvirus-like DNA sequences. *N. Engl. J. Med.* **1996**, *334*, 272–273. https://doi.org/10.1056/NEJM199601253340417.
3. Carbone, A.; Gloghini, A.; Vaccher, E.; Zagonel, V.; Pastore, C.; Dalla Palma, P.; Branz, F.; Saglio, G.; Volpe, R.; Tirelli, U.; et al. Kaposi's sarcoma-associated herpesvirus DNA sequences in AIDS-related and AIDS-unrelated lymphomatous effusions. *Br. J. Haematol.* **1996**, *94*, 533–543. https://doi.org/10.1046/j.1365-2141.1996.d01-1826.x.
4. Ascoli, V.; Lo Coco, F.; Artini, M.; Levrero, M.; Fruscalzo, A.; Mecucci, C. Primary effusion Burkitt's lymphoma with t(8;22) in a patient with hepatitis C virus-related cirrhosis. *Hum. Pathol.* **1997**, *28*, 101–104. https://doi.org/10.1016/s0046-8177(97)90287-2.
5. Carbone, A.; Cilia, A.M.; Gloghini, A.; Canzonieri, V.; Pastore, C.; Todesco, M.; Cozzi, M.; Perin, T.; Volpe, R.; Pinto, A.; et al. Establishment of HHV-8-positive and HHV-8-negative lymphoma cell lines from primary lymphomatous effusions. *Int. J. Cancer* **1997**, *73*, 562–569. https://doi.org/10.1002/(sici)1097-0215(19971114)73:4<562::aid-ijc18>3.0.co;2-b.
6. Ichinohasama, R.; Miura, I.; Kobayashi, N.; Saitoh, Y.; DeCoteau, J.F.; Saiki, Y.; Mori, S.; Kadin, M.E.; Ooya, K. Herpes virus type 8-negative primary effusion lymphoma associated with PAX-5 gene rearrangement and hepatitis C virus: A case report and review of the literature. *Am. J. Surg. Pathol*. **1998**, *22*, 1528–1537. https://doi.org/10.1097/00000478-199812000-00010.
7. Tanaka, S.; Katano, H.; Tsukamoto, K.; Jin, M.; Oikawa, S.; Nishihara, H.; Sawa, H.; Sawada, K.; Shimizu, M.; Sata, T.; et al. HHV8-negative primary effusion lymphoma of the peritoneal cavity presenting with a distinct immunohistochemical phenotype. *Pathol. Int.* **2001**, *51*, 293–300. https://doi.org/10.1046/j.1440-1827.2001.01189.x.
8. Ashihara, E.; Shimazaki, C.; Hirai, H.; Inaba, T.; Hasegawa, G.; Mori, S.; Nakagawa, M. Human herpes virus 8-negative primary effusion lymphoma in a patient with a ventriculoperitoneal shunt tube. *Int. J. Hematol.* **2001**, *74*, 327–332. https://doi.org/10.1007/BF02982069.
9. Hara, T.; Nishi, S.; Horimoto, A.; Takenaka, S.; Ibata, Y.; Akamatsu, H. Primary effusion lymphoma in a patient with hepatitis C virus-related liver cirrhosis. *J. Gastroenterol. Hepatol*. **2001**, *16*, 948–949.
10. Ohori, N.P.; Whisnant, R.E.; Nalesnik, M.A.; Swerdlow, S.H. Primary pleural effusion posttransplant lymphoproliferative disorder: Distinction from secondary involvement and effusion lymphoma. *Diagn. Cytopathol.* **2001**, *25*, 50–53. https://doi.org/10.1002/dc.2001.
11. Rodriguez, J.; Romaguera, J.E.; Katz, R.L.; Said, J.; Cabanillas, F. Primary effusion lymphoma in an HIV-negative patient with no serologic evidence of Kaposi's sarcoma virus. *Leuk. Lymphoma.* **2001**, *41*, 185–189. https://doi.org/10.3109/10428190109057969.
12. Yamamoto, Y.; Kitajima, H.; Sakihana, H.; Shigeki, T.; Fukuhara, S. CD3+CD4-CD8-TCR-alphabeta+ T-cell lymphoma with clinical features of primary effusion lymphoma: An autopsy case. *Int. J. Hematol.* **2001**, *74*, 442–446. https://doi.org/10.1007/BF02982089.
13. Ohshima, K.; Ishiguro, M.; Yamasaki, S.; Miyagi, J.; Okamura, S.; Sugio, Y.; Muta, T.; Sasaki, H.; Tuchiya, T.; Kawasaki, C.; et al. Chromosomal and comparative genomic analyses of HHV-8-negative primary effusion lymphoma in five HIV-negative Japanese patients. *Leuk. Lymphoma* **2002**, *43*, 595–601. https://doi.org/10.1080/10428190290012100.
14. Hisamoto, A.; Yamane, H.; Hiraki, A.; Maeda, Y.; Fujii, N.; Sasaki, K.; Miyake, T.; Sasaki, T.; Nakamura, T.; Kiura, K.; et al. Human herpes virus-8-negative primary effusion lymphoma in a patient with common variable immunodeficiency. *Leuk. Lymphoma* **2003**, *44*, 2019–2022. https://doi.org/10.1080/1042819031000110955.
15. Chiba, H.; Matsunaga, T.; Kuribayashi, K.; Nikaido, T.; Shirao, S.; Murakami, K.; Hirayama, Y.; Sakamaki, S.; Ikeda, H.; Niitsu, Y. Autoimmune hemolytic anemia as a first manifestation of primary effusion lymphoma. *Ann. Hematol*. **2003**, *82*, 773–776. https://doi.org/10.1007/s00277-003-0734-x.
16. Nakamura, Y.; Tajima, F.; Omura, H.; Ishiga, K.; Kawatani, T.; Murawaki, Y. Primary effusion lymphoma of the left scrotum. *Intern. Med.* **2003**, *42*, 351–353. https://doi.org/10.2169/internalmedicine.42.351.
17. Paner, G.P.; Jensen, J.; Foreman, K.E.; Reyes, C.V. HIV and HHV-8 negative primary effusion lymphoma in a patient with hepatitis C virus-related liver cirrhosis. *Leuk. Lymphoma* **2003**, *44*, 1811–1814. https://doi.org/10.1080/1042819031000104015.
18. Shimazaki, M.; Fujita, M.; Tsukamoto, K.; Matsuki, T.; Iwata, M.; Takahashi, H.; Doi, A.; Hyakkoku, M.; Yamauchi, K.; Genda, S.; et al. An unusual case of primary effusion lymphoma in a HIV-negative patient not pathogenetically associated with HHV8. *Eur. J. Haematol.* **2003**, *71*, 62–67. https://doi.org/10.1034/j.1600-0609.2003.00083.x.
19. Inoue, Y.; Tsukasaki, K.; Nagai, K.; Soda, H.; Tomonaga, M. Durable remission by sobuzoxane in an HIV-seronegative patient with human herpesvirus 8-negative primary effusion lymphoma. *Int. J. Hematol.* **2004**, *79*, 271–275. https://doi.org/10.1532/ijh97.03107.
20. Nonami, A.; Yokoyama, T.; Takeshita, M.; Ohshima, K.; Kubota, A.; Okamura, S. Human herpes virus 8-negative primary effusion lymphoma (PEL) in a patient after repeated chylous ascites and chylothorax. *Intern. Med.* **2004**, *43*, 236–242. https://doi.org/10.2169/internalmedicine.43.236.
21. Takao, T.; Kobayashi, Y.; Kuroda, J.; Omoto, A.; Nishimura, T.; Kamitsuji, Y.; Fukiya, E.; Nakamura, C.; Kimura, S.; Yoshikawa, T. Rituximab is effective for human herpesvirus-8-negative primary effusion lymphoma with CD20 phenotype associated hepatitis C virus-related liver cirrhosis. *Am. J. Hematol.* **2004**, *77*, 419–420. https://doi.org/10.1002/ajh.20227.

22. Fujiwara, T.; Ichinohasama, R.; Miura, I.; Sugawara, T.; Harigae, H.; Yokoyama, H.; Takahashi, S.; Tomiya, Y.; Yamada, M.; Ishizawa, K.; et al. Primary effusion lymphoma of the pericardial cavity carrying t(1;22)(q21;q11) and t(14;17)(q32;q23). *Cancer Genet*. *Cytogenet*. **2005**, *156*, 49–53. https://doi.org/10.1016/j.cancergencyto.2004.04.013.
23. Jenkins, C.; Sorour, Y.; Blake, E.; Elliot, R.; Al-Sabah, A.I.; Green, J. Human-immunodeficiency-virus-negative, human-herpes-virus-8-negative abdominal cavity primary effusion lymphoma. *Clin. Oncol.* **2005**, *17*, 636–638. https://doi.org/10.1016/j.clon.2005.05.012.
24. Matsumoto, Y.; Nomura, K.; Ueda, K.; Satoh, K.; Yasuda, N.; Taki, T.; Yokota, S.; Horiike, S.; Okanoue, T.; Taniwaki, M. Human herpesvirus 8-negative malignant effusion lymphoma: A distinct clinical entity and successful treatment with rituximab. *Leuk. Lymphoma* **2005**, *46*, 415–419. https://doi.org/10.1080/10428190400018364.
25. Venizelos, I.; Tamiolakis, D.; Lambropoulou, M.; Nikolaidou, S.; Bolioti, S.; Papadopoulos, H.; Papadopoulos, N. An unusual case of posttransplant peritoneal primary effusion lymphoma with T-cell phenotype in a HIV-negative female, not associated with HHV-8. *Pathol. Oncol. Res.* **2005**, *11*, 178–181. https://doi.org/10.1007/BF02893396.
26. Youngster, I.; Vaisben, E.; Cohen, H.; Nassar, F. An unusual cause of pleural effusion. *Age Ageing* **2006**, *35*, 94–96. https://doi.org/10.1093/ageing/afj009.
27. Kobayashi, Y.; Kamitsuji, Y.; Kuroda, J.; Tsunoda, S.; Uoshima, N.; Kimura, S.; Wada, K.; Matsumoto, Y.; Nomura, K.; Horiike, S.; et al. Comparison of human herpes virus 8 related primary effusion lymphoma with human herpes virus 8 unrelated primary effusion lymphoma-like lymphoma on the basis of HIV: Report of 2 cases and review of 212 cases in the literature. *Acta Haematol.* **2007**, *117*, 132–144. https://doi.org/10.1159/000097460.
28. Niino, D.; Tsukasaki, K.; Torii, K.; Imanishi, D.; Tsuchiya, T.; Onimaru, Y.; Tsushima, H.; Yoshida, S.; Yamada, Y.; Kamihira, S.; et al. Human herpes virus 8-negative primary effusion lymphoma with BCL6 rearrangement in a patient with idiopathic CD4 positive T-lymphocytopenia. *Haematologica* **2008**, *93*, e21–e23. https://doi.org/10.3324/haematol.12085.
29. Terasaki, Y.; Okumura, H.; Saito, K.; Sato, Y.; Yoshino, T.; Ichinohasama, R.; Ishida, Y. HHV-8/KSHV-negative and CD20-positive primary effusion lymphoma successfully treated by pleural drainage followed by chemotherapy containing rituximab. *Intern. Med.* **2008**, *47*, 2175–2178. https://doi.org/10.2169/internalmedicine.47.1565.
30. Adiguzel, C.; Bozkurt, S.U.; Kaygusuz, I.; Uzay, A.; Tecimer, T.; Bayik, M. Human herpes virus 8-unrelated primary effusion lymphoma-like lymphoma: Report of a rare case and review of the literature. *APMIS* **2009**, *117*, 222–229. https://doi.org/10.1111/j.1600-0463.2008.00005.x.
31. De Filippi, R.; Iaccarino, G.; Frigeri, F.; Di Francia, R.; Crisci, S.; Capobianco, G.; Arcamone, M.; Becchimanzi, C.; Amoroso, B.; De Chiara, A.; et al. Elevation of clonal serum free light chains in patients with HIV-negative primary effusion lymphoma (PEL) and PEL-like lymphoma. *Br. J. Haematol.* **2009**, *147*, 405–408. https://doi.org/10.1111/j.1365-2141.2009.07846.x.
32. Tsagarakis, N.J.; Argyrou, A.; Gortzolidis, G.; Kentrou, N.; Papadhimitriou, S.I.; Tzanetou, K.; Kakiopoulos, G.; Papadimitriou, K.A.; Skoumi, D.; Paterakis, G. Report of an HIV and HHV-8 negative case of primary effusion lymphoma with idiopathic T4 lymphocytopenia. *Int. J. Hematol*. **2009**, *90*, 94–98. https://doi.org/10.1007/s12185-009-0343-0.
33. Takahashi, T.; Hangaishi, A.; Yamamoto, G.; Ichikawa, M.; Imai, Y.; Kurokawa, M. HIV-negative, HHV-8-unrelated primary effusion lymphoma-like lymphoma: Report of two cases. *Am. J. Hematol*. **2010**, *85*, 85–87. https://doi.org/10.1002/ajh.21568.
34. Cooper, A.R.; Burack, W.R.; Allerton, J.P. A case of Kaposi sarcoma-associated herpesvirus/human herpesvirus 8-unrelated but Epstein-Barr virus-positive primary effusion lymphoma-like lymphoma in the setting of human immunodeficiency virus and hepatitis C virus infection. *Leuk. Lymphoma* **2010**, *51*, 2303–2305. https://doi.org/10.3109/10428194.2010.520775.
35. Kagoya, Y.; Takahashi, T.; Yoshimoto, T.; Ichikawa, M.; Hangaishi, A.; Fukayama, M.; Kurokawa, M. Recurrent pericardial effusion after treatment for primary effusion lymphoma-like lymphoma: An autopsied case. *Ann. Hematol*. **2011**, *90*, 219–220. https://doi.org/10.1007/s00277-010-0975-4.
36. Terasaki, Y.; Yamamoto, H.; Kiyokawa, H.; Okumura, H.; Saito, K.; Ichinohasama, R.; Ishida, Y. Disappearance of malignant cells by effusion drainage alone in two patients with HHV-8-unrelated HIV-negative primary effusion lymphoma-like lymphoma. *Int. J. Hematol.* **2011**, *94*, 279–284. https://doi.org/10.1007/s12185-011-0906-8.
37. Wang, T.; Nava, V.E.; Schechter, G.P.; Lichy, J.H.; Liu, M.L. Human herpes virus 8-unrelated primary effusion lymphoma-like lymphoma: A patient successfully treated with pleurodesis. *J. Clin. Oncol.* **2011**, *29*, e747–e750. https://doi.org/10.1200/JCO.2011.35.7509.
38. Sumida, K.; Ubara, Y.; Takaichi, K.; Wake, A. Primary effusion lymphoma-like lymphoma with polycystic kidney disease. *BMJ Case Rep.* **2012**, *2012*, bcr0220125774. https://doi.org/10.1136/bcr-02-2012-5774.
39. Kim, K.H.; Lee, J.H.; Jeong, H.C.; Kim, G.W.; Song, S.H.; Jung, S.Y.; Kim, G.I.; Kim, E.K. A case of human herpes virus-8 unrelated primary effusion lymphoma-like lymphoma presented as pleural effusion. *Tuberc. Respir. Dis.* **2012**, *73*, 336–341. https://doi.org/10.4046/trd.2012.73.6.336.
40. Alexanian, S.; Said, J.; Lones, M.; Pullarkat, S.T. KSHV/HHV8-negative effusion-based lymphoma, a distinct entity associated with fluid overload states. *Am. J. Surg. Pathol.* **2013**, *37*, 241–249. https://doi.org/10.1097/PAS.0b013e318267fabc.
41. Wu, W.; Youm, W.; Rezk, S.A.; Zhao, X. Human herpesvirus 8-unrelated primary effusion lymphoma-like lymphoma: Report of a rare case and review of 54 cases in the literature. *Am. J. Clin. Pathol.* **2013**, *140*, 258–273. https://doi.org/10.1309/ajcphz3cho4huwet.
42. Xiao, J.; Selvaggi, S.M.; Leith, C.P.; Fitzgerald, S.A.; Stewart, J., 3rd. Kaposi sarcoma herpesvirus/human herpesvirus-8-negative effusion-based lymphoma: Report of 3 cases and review of the literature. *Cancer Cytopathol.* **2013**, *121*, 661–669. https://doi.org/10.1002/cncy.21311.

43. Mohammad, F.; Siddique, M.N.; Siddiqui, F.; Popalzai, M.; Asgari, M.; Odaimi, M. A Unique Case of Malignant Pleuropericardial Effusion: HHV-8-Unrelated PEL-Like Lymphoma-A Case Report and Review of the Literature. *Case Rep. Oncol. Med.* **2014**, *2014*, 436821. https://doi.org/10.1155/2014/436821.
44. Fan, H.B.; Yang, D.L.; Guo, Y.; Chen, A.S.; Zhou, M.X.; Wu, J.J.; Ma, X.J.; Li, Z. Human herpes virus 8-unrelated primary effusion lymphoma-like lymphoma in a patient with hepatitis B virus-related liver cirrhosis: A case report. *J. Res. Med. Sci.* **2014**, *19*, 190–192.
45. Choi, J.W.; Kim, Y.; Lee, J.H.; Kim, Y.S. Human Herpesvirus 8-Negative and Epstein-Barr Virus-Positive Effusion-Based Lymphoma in a Patient with Human Immunodeficiency Virus. *J. Pathol. Transl. Med.* **2015**, *49*, 409–412. https://doi.org/10.4132/jptm.2015.06.03.
46. Usmani, A.; Walts, A.E.; Patel, S.; Alkan, S.; Kitahara, S. HHV8-negative effusion based lymphoma: A series of 17 cases at a single institution. *J. Am. Soc. Cytopathol.* **2015**, *4*, 37–43. https://doi.org/10.1016/j.jasc.2014.10.002.
47. Shin, J.; Lee, J.O.; Choe, J.Y.; Bang, S.M.; Lee, J.S. Human Herpesvirus 8-Unrelated Primary Effusion Lymphoma-Like Lymphoma in an Elderly Korean Patient with a Good Response to Rituximab Plus Cyclophosphamide, Doxorubicin, Vincristine, and Prednisolone. *Cancer Res. Treat.* **2017**, *49*, 274–278. https://doi.org/10.4143/crt.2016.076.
48. Chen, B.J.; Wang, R.C.; Ho, C.H.; Yuan, C.T.; Huang, W.T.; Yang, S.F.; Hsieh, P.P.; Yung, Y.C.; Lin, S.Y.; Hsu, C.F.; et al. Primary effusion lymphoma in Taiwan shows two distinctive clinicopathological subtypes with rare human immunodeficiency virus association. *Histopathology* **2018**, *72*, 930–944. https://doi.org/10.1111/his.13449.
49. Kaji, D.; Ota, Y.; Sato, Y.; Nagafuji, K.; Ueda, Y.; Okamoto, M.; Terasaki, Y.; Tsuyama, N.; Matsue, K.; Kinoshita, T.; et al. Primary human herpesvirus 8-negative effusion-based lymphoma: A large B-cell lymphoma with favorable prognosis. *Blood. Adv.* **2020**, *4*, 4442–4450. https://doi.org/10.1182/bloodadvances.2020002293.
50. Kim, M.; An, J.; Yoon, S.O.; Yong, S.H.; Kim, J.S.; Yang, W.I.; Leem, A.Y. Human herpesvirus 8-negative effusion-based lymphoma with indolent clinical behavior in an elderly patient: A case report and literature review. *Oncol. Lett.* **2020**, *20*, 343. https://doi.org/10.3892/ol.2020.12206.
51. Fiori, S.; Todisco, E.; Ramadan, S.; Gigli, F.; Falco, P.; Iurlo, A.; Rampinelli, C.; Croci, G.; Pileri, S.A.; Tarella, C. HHV8-Negative Effusion-Based Large B Cell Lymphoma Arising in Chronic Myeloid Leukemia Patients under Dasatinib Treatment: A Report of Two Cases. *Biology* **2021**, *10*. 152.https://doi.org/10.3390/biology10020152.
52. Wang, R.C.; Chen, Y.H.; Chen, B.J.; Chuang, S.S. The cytopathological spectrum of lymphomas in effusions in a tertiary center in Taiwan. *Diagn. Cytopathol.* **2021**, *49*, 232–240. https://doi.org/10.1002/dc.24626.
